# Do successful PhD outcomes reflect the research environment rather than academic ability?

**DOI:** 10.1371/journal.pone.0236327

**Published:** 2020-08-05

**Authors:** Daniel L. Belavy, Patrick J. Owen, Patricia M. Livingston

**Affiliations:** 1 Institute for Physical Activity and Nutrition (IPAN), School of Exercise and Nutrition Sciences, Deakin University, Geelong, Victoria, Australia; 2 Faculty of Health, Office of Faculty of Health, Deakin University, Geelong, Victoria, Australia; Universitat de Barcelona, SPAIN

## Abstract

Maximising research productivity is a major focus for universities world-wide. Graduate research programs are an important driver of research outputs. Choosing students with the greatest likelihood of success is considered a key part of improving research outcomes. There has been little empirical investigation of what factors drive the outcomes from a student's PhD and whether ranking procedures are effective in student selection. Here we show that, the research environment had a decisive influence: students who conducted research in one of the University's priority research areas and who had experienced, research-intensive, supervisors had significantly better outcomes from their PhD in terms of number of manuscripts published, citations, average impact factor of journals published in, and reduced attrition rates. In contrast, students’ previous academic outcomes and research training was unrelated to outcomes. Furthermore, students who received a scholarship to support their studies generated significantly more publications in higher impact journals, their work was cited more often and they were less likely to withdraw from their PhD. The findings suggest that experienced supervisors researching in a priority research area facilitate PhD student productivity. The findings question the utility of assigning PhD scholarships solely on the basis of student academic merit, once minimum entry requirements are met. Given that citations, publication numbers and publications in higher ranked journals drive university rankings, and that publications from PhD student contribute approximately one-third of all research outputs from universities, strengthening research infrastructure and supervision teams may be more important considerations for maximising the contribution of PhD students to a university’s international standing.

## Introduction

A research doctorate degree comprises a process of independent research that produces an original contribution to knowledge [1]. The Australian Commonwealth Government supports [2] both domestic and overseas students undertaking research doctorate degrees, known as PhDs. These scholarships, which comprise a stipend for three years, are competitive. For this reason, when students apply for scholarships for their PhD studies, prior academic performance and research training play a key role in deciding whether the applicant receives a scholarship. However, is assigning scholarships predominately on the basis of academic grades and previous research experience effective in determining who will succeed?

A university’s international and national ranking is important for its reputation and marketing to prospective students [3]. Citation rates, number of publications and impact factor of journals faculty publish in, influence the ranking of a university. The Quacquarelli Symonds University Rank [4] is weighted 30% by the number of citations per faculty member, the Times Higher Education World Ranking [5] 30% by the number of citations and 6% by the number of publications per academic, and the Academic Ranking of World Universities [6] 20% by number of highly cited researchers, 20% by number of papers published in Nature or Science and 20% by the number of publications in total.

PhD students are important drivers of research outputs from universities, with one analysis [7] showing that one-third of research publications was from doctoral students. It is important to consider to what extent the procedures by which universities select students who go on to produce higher numbers of highly cited publications in high impact journals. We are not aware of any prior research that has examined this topic.

Waldinger [8] showed that the quality of academic staff (in departments of mathematics at German universities in the 1930s) influenced the likelihood of whether a doctoral student would become a full professor later in their career. Waldinger also showed that the amount of citations the scientific work of a doctoral student received through their entire subsequent scientific career was influenced by the status of their supervisor. Other factors, such as, the reputation of a department [9], the reputation the group leader [10], and access to resources and equipment [11], the number of full-professors on staff [12] influenced the research output of the academics involved in that group. Less information is available on the impact of student academic ability or prior research training on PhD outcomes: one analysis found that the reputation of a given department was more important for employment outcomes post-PhD than the accomplishments of the student during their studies [13]. Overall, the evidence available implies that the research environment may have an inordinate impact on the PhD student outcomes (e.g. citations, number of publications, impact factor of journals of those publications).

Here we examine the relationship between information known about applicants and their proposed supervisory teams at the time of scholarship application with the subsequent research outputs, as measured by number of citations, number of publications and the impact of journals of those publications.

## Materials and methods

Deakin University Human Research Ethics Committee reviewed this project (2019–191) and found it to be compliant with the Ethical Considerations in Quality Assurance and Evaluation Activities guidelines of the National Health and Medical Research Council of Australia and determined that no further ethics review was required. Consent was not obtained and the data analysed anonymously.

**Fig 1:**
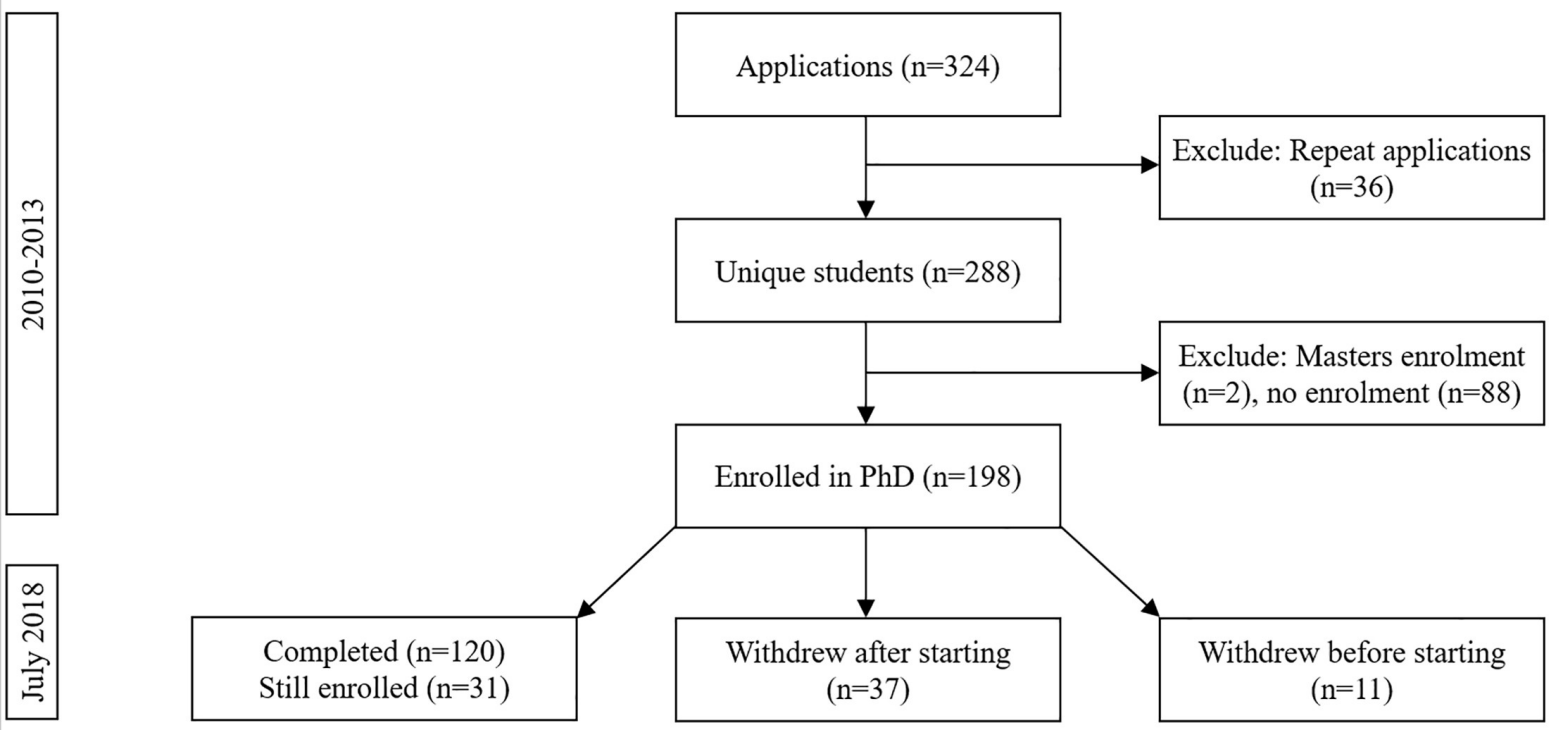
Data set and student completions. In 2010 to 2013, applications were submitted for PhD scholarships and in July 2018 data on publication outputs and completion of degree were obtained. Overall, 11 students did not enrol in PhD despite an offer with scholarship being made and 37 withdrew from their studies after starting.

Over a four year period, 2010–2013, 324 PhD scholarship applications were submitted to the Faculty of Health at one university in Australia (Fig 1). In these applications, data were collated on:

- the grade the student achieved for their prior research training degree and their rank in this degree (top, middle, bottom third of first class honours or second class honours; or their equivalency to this),
- the grade point average achieved in their undergraduate degree (ranked on a scale of 1 to 5 with 5 = high distinction grade point average plus prizes awarded, 4 = high distinction grade point average, 3 = distinction, 2 = credit, 1 = pass).
- whether the applicant had published in a scientific journal (‘yes’ or ‘no’)
- research environment: whether the primary supervisor was located in a strategic research centre or institute within the university (‘yes’ or ‘no’).

At the time of ranking for scholarships, the review panel scored each application on the basis of their academic merit and the research experience, alignment of the proposed research with the strategic research goals of the Faculty and university, and the experience of the supervisory team (as expressed by prior PhD completions, student progress, external grants, previous student publications, supervisor track record). In July 2018, these scores were reviewed by two independent assessors experienced in the scholarship ranking process and consensus was attained. Subsequent to this, following variables were generated:

- quartile of the academic merit scores in which each student was located.
- strategic alignment score achieved maximum points (‘yes’ or ‘no’). The presence or absence of a maximum score was taken for this variable as there were few instances of low scores on this criterion and data were skewed to the maximum score.
- supervisor team scores achieved maximum points (‘yes’ or ‘no’). The presence or absence of maximum score was taken for this variable as there were few instances of low scores on this criterion and data were skewed to the maximum score.
- level of academic appointment of the primary supervisor (lecturer/senior lecturer, associate professor, or full professor)

Data on whether the applicant subsequently enrolled (if ‘no’ they were excluded from further analysis; Fig 1), whether they completed their studies (‘yes’ or ‘no’), and whether the student received a scholarship to support his/her study (‘yes’ or ‘no’) obtained from another university database.

The university tracks publication outputs of its faculty and students. In July 2018, these data were obtained to link the number of publications by the student with their primary supervisor, the impact factor of the journals in which these publications appeared, and the number of citations received by the publications in Web of Science by the cut-off data of data access. Publications were matched on the basis of student name and primary supervisor name. If a change of primary supervisor occurred during student candidature, publication matches with the new primary supervisor were included as well. If the student had enrolled in a PhD but achieved no publications within the time-period examined, data were coded as zero publications, zero citations and zero average impact factor. Datasets were merged in using custom written code implemented in the 'R' statistical environment (version 3.4.0 https://www.r-project.org/). Where repeat applications were submitted in subsequent years by the same person, only the data available at the first application was used in further analysis. Prior to statistical analysis, all identifying information was removed.

### Statistical analyses

All analyses were conducted using Stata statistical software version 15 (College Station TX, USA). Univariate associations between continuous dependent variables (number of publications, number of citations, number of citations per publication, average publication impact factor) and explanatory variables were assessed by the Kruskal-Wallis H test or Mann-Whitney U test (both non-parametric tests), as well as one-way analysis of variance and t-tests (both parametric tests). Univariate associations between withdrawal (yes/no) and independent variables were assessed by penalized maximum likelihood [14,15] logistic regression. We categorised the explanatory variables as follows: student specific factors (student research degree rank, student undergraduate rank, student prior publication, student academic merit), supervisor specific factors (supervisor located in a strategic research centre, supervisor academic level, supervisor team scores achieved maximum points), research topic related factors (strategic alignment score achieved maximum points), and whether a scholarship was awarded. To investigate which variables were more important than others for PhD student outcome metrics, factorial analysis of variance (ANOVA) as well as stepwise multiple linear regression models with both forward and backward selection were used to assess the association between the dependent variables and the independent variables. We further conducted factorial ANOVA to assess the association between the dependent variables and independent variables. Stepwise penalized maximum likelihood logistic regression models were used to predict withdrawal from PhD (yes/no) based on independent variables. An adjusted alpha level of 0.10 to enter and 0.20 to remove were used for all step-wise regression models. An alpha-level of 0.05 was adopted for all other statistical tests, including the assessment of the final step-wise regression models.

## Results

Primary analyses involved 198 students who enrolled in PhD (61% of 324 applications; Fig 1). The descriptive data on the characteristics of the students are shown in Table 1. In the whole cohort, median (25^th^ percentile, 75^th^ percentile) and mean (standard deviation; SD) number of publications were 1.0 (0.0, 3.0) and 2.8 (4.4), impact factor 0.86 (0.00, 2.61) and 1.59 (2.36), citations per publication 0.0 (0.0, 4.5) and 3.5 (7.4) and total citations 0.0 (0.0, 17.0) and 19.6 (49.8). S1 Table presents the stability of the explanatory variables across each year of student applications. The relationship between ranking criteria and PhD student output metrics are shown in Table 2 (non-parametric analyses) and Table 3 (parametric analyses). Findings of both non-parametric and parametric analyses were similar. Non-parametric (S1 Table) and parametric (S2 Table) effect sizes as well as variability among variables by year of application (S3 Table) are reported in the data supplement.

**Table 1:** Descriptive data for the ranking criteria of the 198 unique PhD applications and risk of withdrawing from PhD.

Variable	N (%)	Withdrawing from PhD	
		Odds ratio	Relative risk
Student research training degree (n = 183)		1.23 (P = 0.179)	
1st class honours, top tertile	90 (49.2)		1.00
1st class honours, middle tertile	34 (18.6)		0.66 (0.24, 1.84)
1st class honours, lower tertile	24 (13.1)		0.70 (0.22, 2.22)
2nd class honours	35 (19.1)		1.77 (0.91, 3.32)
Student undergraduate grades (n = 167)		0.77 (P = 0.288)	
Grade point average ≥80% plus prizes awarded	14 (8.4)		1.00
Grade point average ≥80%	46 (27.5)		0.68 (0.25, 1.89)
Grade point average ≥70%, but less than 80%	79 (47.3)		0.44 (0.16, 1.22)
Grade point average ≥60%, but less than 70%	28 (16.8)		0.63 (0.20, 1.97)
Student had prior publication (n = 194)		0.93 (P = 0.864)	
Yes	51 (26.3)		1.00
No	143 (73.7)		0.96 (0.50, 1.85)
Student academic merit (n = 198)		1.30 (P = 0.110)	
1^st^ quartile	48 (24.3)		1.00
2^nd^ quartile	50 (25.3)		1.10 (0.43, 2.79)
3^rd^ quartile	50 (25.3)		1.10 (0.43, 2.79)
4^th^ quartile	50 (25.3)		1.92 (0.85, 4.34)
Supervisor in strategic research centre (n = 198)		1.34 (P = 0.46)	
Yes	143 (72.2)		1.00
No	55 (27.8)		1.25 (0.68, 2.31)
Supervisor academic level at application (n = 184)		1.32 (P = 0.216)	
Full-professor	53 (28.8)		1.00
Associate professor	52 (28.3)		0.91 (0.38, 2.17)
Senior lecturer or lecturer	79 (42.9)		1.49 (0.74, 3.02)
Alignment of research achieved maximum score (n = 198)		**2.88 (P = 0.004)**	
Yes	165 (83.3)		1.00
No	33 (16.7)		**2.35 (1.31, 4.20)**
Supervisory team achieved maximum score (n = 198)		1.95 (P = 0.126)	
Yes	127 (64.1)		1.00
No	71 (35.9)		1.66 (0.87, 3.17)
Scholarship awarded (n = 198)		**3.04 (P = 0.006)**	
Yes	90 (45.5)		1.00
No	108 (54.6)		**2.59 (1.29, 5.21)**

Number of students for which each variable was available is indicted in brackets following variable. Data for each level of each variable are count (percentage of available data). Data for withdrawal from PhD are odds ratio (P-value) in second column from right for the parameter overall and relative risk (95% confidence interval) compared to the reference level in the right column. Significant risk ratios are bolded.

**Table 2:** Non-parametric analyses: Associations between the ranking criteria of the 198 unique PhD applications and researcher metrics.

Variable	Number of publications	Number of citations	Number of citations per publication	Average impact factor
Student research training degree	P = 0.361	P = 0.383	P = 0.429	P = 0.199
1st class honours, top	2.0 (0.0, 4.0)	2.0 (0.0, 19.0)	1.0 (0.0, 3.9)	1.3 (0.0, 3.0)
1st class honours, middle	1.0 (0.0, 3.0)	2.0 (0.0, 18.0)	1.0 (0.0, 5.9)	1.0 (0.0, 2.5)
1st class honours, lower	1.0 (0.0, 3.0)	0.0 (0.0, 10.0)	0.0 (0.0, 3.3)	0.3 (0.0, 2.4)
2nd class honours	0.0 (0.0, 3.0)	0.0 (0.0, 30.0)	0.0 (0.0, 9.0)	0.0 (0.0, 2.2)
Student undergraduate rank	P = 0.588	P = 0.668	P = 0.643	P = 0.420
GPA≥80% plus prizes	2.0 (0.0, 3.0)	3.0 (0.0, 19.0)	1.2 (0.0, 6.0)	2.2 (0.0, 3.0)
GPA≥80%	1.0 (0.0, 3.0)	0.0 (0.0, 9.0)	0.0 (0.0, 3.4)	0.8 (0.0, 3.0)
GPA≥70% and <80%	2.0 (0.0, 4.0)	1.0 (0.0, 18.0)	0.3 (0.0, 4.0)	1.0 (0.0, 2.6)
GPA≥60% and <70%	0.5 (0.0, 2.0)	0.0 (0.0, 15.0)	0.0 (0.0, 3.6)	0.0 (0.0, 2.5)
Student had prior publication	P = 0.551	P = 0.436	P = 0.545	P = 0.809
Yes	1.0 (0.0, 4.0)	1.0 (0.0, 20.0)	0.7 (0.0, 4.5)	1.1 (0.0, 2.3)
No	1.0 (0.0, 3.0)	0.0 (0.0, 14.0)	0.0 (0.0, 4.5)	0.7 (0.0, 2.8)
Student academic merit	**P = 0.017**	P = 0.080	P = 0.082	**P = 0.001**
1^st^ quartile	2.0 (1.0, 5.0)^d^	4.0 (0.0, 19.0)	1.7 (0.0, 5.2)	2.2 (0.6, 3.3)^d^#
2^nd^ quartile	0.5 (0.0, 4.0)	0.0 (0.0, 17.0)	0.0 (0.0, 3.9)	0.0 (0.0, 2.3)
3^rd^ quartile	1.0 (0.0, 3.0)	0.0 (0.0, 29.0)	0.0 (0.0, 5.0)	0.9 (0.0, 2.4)
4^th^ quartile	0.0 (0.0, 2.0)	0.0 (0.0, 10.0)	0.0 (0.0, 3.0)	0.0 (0.0, 2.2)
Supervisor in institute or research centre	**P<0.001**	**P<0.001**	**P<0.001**	**P<0.001**
Yes	2.0 (0.0, 4.0)^c^†	3.0 (0.0, 23.0)^c^†	1.0 (0.0, 5.7)^c^†	1.4 (0.0, 3.0)^c^†
No	0.0 (0.0, 2.0)	0.0 (0.0, 2.0)	0.0 (0.0, 1.0)	0.0 (0.0, 1.0)
Supervisor academic level at application	P = 0.107	P = 0.482	P = 0.524	P = 0.125
Full-professor	2.0 (0.0, 4.0)	3.0 (0.0, 18.0)	1.0 (0.0, 4.8)	1.2 (0.0, 2.6)
Associate professor	1.0 (0.0, 3.5)	1.5 (0.0, 18.0)	1.0 (0.0, 4.4)	1.2 (0.0, 3.0)
Senior lecturer or lecturer	0.0 (0.0, 3.0)	0.0 (0.0, 12.0)	0.0 (0.0, 3.9)	0.0 (0.0, 2.5)
Supervisory team achieved maximum score	**P<0.001**	**P = 0.003**	**P<0.001**	**P<0.001**
Yes	2.0 (0.0, 5.0)^c^*	4.0 (0.0, 20.0)^b^*	1.5 (0.0, 5.9)^c^	1.5 (0.0, 3.0)^c^†
No	0.0 (0.0, 2.0)	0.0 (0.0, 3.0)	0.0 (0.0, 1.0)	0.0 (0.0, 2.1)
Alignment of research achieved maximum score	P = 0.273	P = 0.233	P = 0.146	P = 0.123
Yes	1.0 (0.0, 3.0)	1.0 (0.0, 18.0)	1.0 (0.0, 4.8)	1.0 (0.0, 2.8)
No	0.0 (0.0, 3.5)	0.0 (0.0, 11.0)	0.0 (0.0, 3.2)	0.0 (0.0, 2.1)
Scholarship awarded	**P<0.001**	**P<0.001**	**P<0.001**	**P<0.001**
Yes	2.0 (1.0, 6.0)^c^‡	8.5 (0.0, 35.0)^c^†	2.4 (0.0, 5.8)^c^*	2.2 (0.7, 3.2)^c^‡
No	0.0 (0.0, 2.0)	0.0 (0.0, 6.0)	0.0 (0.0, 1.4)	0.0 (0.0, 1.7)

Data for publication numbers, citations and impact factors are median (25^th^ percentile, 75^th^ percentile). For these variables, significance of difference is indicated by ^a^ P<0.05, ^b^ P<0.01, ^c^ P<0.001 compared to ‘no’ and ^d^ P<0.05 compared to all other quartiles. P-values next to parameter name are from the Kruskal-Wallis one-way analysis of variance test. S2 Table presents the effect sizes for these analyses.

**Table 3:** Parametric analyses: Associations between the ranking criteria of the 198 unique PhD applications and researcher metrics.

Variable	Number of publications	Number of citations	Number of citations per publication	Average impact factor
Student research training degree	P = 0.522	P = 0.237	P = 0.747	P = 0.240
1st class honours, top	3.2 (4.4)	19.6 (41.4)	3.3 (5.3)	1.96 (2.95)
1st class honours, middle	2.5 (3.4)	14.9 (26.1)	3.3 (4.6)	1.35 (1.46)
1st class honours, lower	1.8 (2.5)	11.0 (29.4)	4.1 (14)	1.49 (2.09)
2nd class honours	3.1 (6.6)	35.6 (90.2)	4.8 (9.2)	1.06 (1.44)
Student undergraduate rank	P = 0.652	P = 0.994	P = 0.640	P = 0.077
GPA≥80% plus prizes	2.8 (3.5)	17.7 (35.1)	4.2 (6.1)	3.18 (5.65)
GPA≥80%	2.1 (3)	15.3 (32.0)	3.1 (5.2)	1.42 (1.74)
GPA≥70% and <80%	3.1 (4.6)	16.4 (38.1)	2.5 (3.7)	1.61 (2.10)
GPA≥60% and <70%	2.5 (5)	17.4 (42.1)	3.4 (6.9)	1.19 (1.61)
Student had prior publication	P = 0.298	P = 0.876	P = 0.792	P = 0.337
Yes	3.4 (5.3)	20.7 (51.1)	3.8 (7.3)	1.32 (1.43)
No	2.6 (4.1)	19.4 (50.1)	3.5 (7.6)	1.70 (2.62)
Student academic merit	P = 0.758	P = 0.952	P = 0.780	**P = 0.005**
1^st^ quartile	3.3 (3.3)	19.7 (33.5)	3.5 (4.5)	2.46 (3.33) #
2^nd^ quartile	2.8 (4.8)	20.1 (51.4)	2.8 (5.1)	1.38 (2.05)
3^rd^ quartile	2.8 (4.4)	21.3 (53.2)	4.4 (8.5)	1.39 (1.51)
4^th^ quartile	2.3 (3.3)	17.2 (60.4)	3.5 (10.7)	1.06 (1.76)
Supervisor in institute or research centre	**P = 0.002**	**P = 0.010**	**P = 0.009**	**P = 0.001**
Yes	3.4 (4.9)†	25.2 (57.1)†	4.4 (8.4)†	1.94 (2.57)†
No	1.3 (2.4)	5.0 (12.6)	1.3 (2.9)	0.71 (1.33)
Supervisor academic level at application	P = 0.166	P = 0.969	P = 0.160	P = 0.156
Full-professor	3.9 (6.0)	21.4 (56.5)	3.2 (4.7)	1.55 (1.53)
Associate professor	2.7 (3.6)	21.1 (48.2)	5.3 (12.2)	2.17 (3.55)
Senior lecturer or lecturer	2.4 (3.9)	19.3 (50.3)	2.7 (4.5)	1.35 (1.89)
Supervisory team achieved maximum score	**P = 0.014**	**P = 0.012**	P = 0.159	**P = 0.005**
Yes	3.4 (4.4)*	26.2 (59.4)*	4.1 (6.3)	1.95 (2.60)†
No	1.8 (4.3)	7.8 (20.1)	2.5 (9.1)	0.97 (1.69)
Alignment of research achieved maximum score	P = 0.862	P = 0.202	P = 0.521	P = 0.336
Yes	2.8 (4.3)	21.7 (53.8)	3.7 (7.3)	1.68 (2.43)
No	2.6 (5.1)	9.1 (16)	2.7 (7.9)	1.20 (1.90)
Scholarship awarded	**P<0.001**	**P = 0.001**	**P = 0.048**	**P<0.001**
Yes	4.2 (4.8)‡	32.1 (66.2)†	4.7 (8.4)*	2.48 (2.92)‡
No	1.6 (3.7)	9.2 (26)	2.6 (6.4)	0.86 (1.39)

Data for publication numbers, citations and impact factors are mean (standard deviation). For these variables, significance of difference is indicated by * P<0.05, † P<0.01, ‡ P<0.001 compared to ‘no’ and # P<0.05 compared to all other quartile. P-values next to parameter name are from one-way analysis of variance test. S3 Table presents the effect sizes for these analyses.

### Number of publications

**Fig 2:**
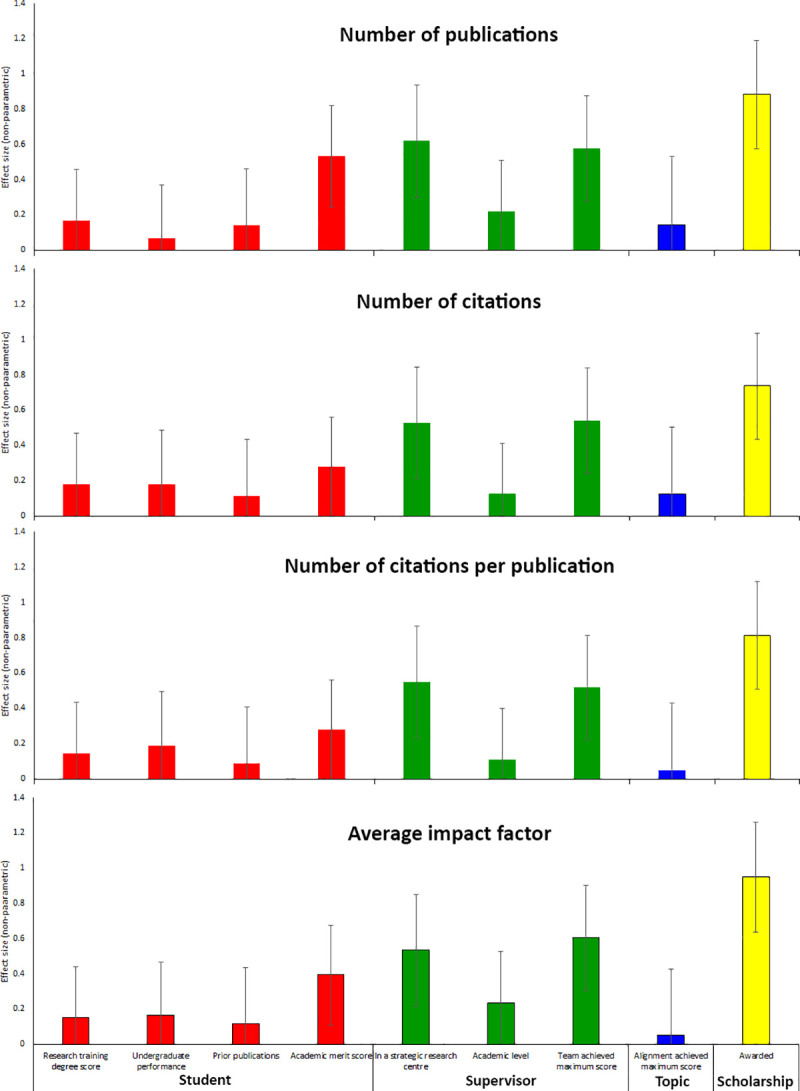
The supervisory team and having a scholarship are the strongest and most consistent factors on outcomes from a PhD. Data are non-parametric effect sizes (95% confidence interval) for each parameter. See S1 Table for more detail and Tables 2 and 3 for more detail on each parameter. Student academic merit score from scholarship panel ranking showed moderate effect sizes, yet these students received 46% of all scholarships and multivariate analyses showed that receiving a scholarship was more important than the student's academic merit (see Results for more detail). Other markers of student ability and prior research training were unrelated to outcomes from the PhD. The score assigned by the panel to the alignment of the research topic with research priorities was unrelated to outcomes.

On univariate analysis (Tables 2 and 3, Fig 2), primary supervisor being located in a strategic research centre (non-parametric and parametric both: P≤0.014), supervisory teams who received a maximum score (both: P≤0.014), being awarded a scholarship (both: P<0.001), student academic merit score (non-parametric: P = 0.017, parametric: P = 0.758) were associated with this outcome, but student undergraduate performance (both: P≥0.588), student research training degree outcome (e.g. first-class honours upper band; both: P≥0.262), research topic (both: P≥0.347), primary supervisor academic level (both: P≥0.107) were not.

Step-wise regression models (Table 4) showed that receiving a scholarship (P = 0.001), primary supervisor being located in a strategic research centre (P = 0.018) remained in final model for number of publications, and whilst 'research topic' remained in the final model, it was not significant (P = 0.076). Factorial ANOVA (S4 Table) yielded similar results (having a scholarship, supervisory teams who received a maximum score, primary supervisor being located in a strategic research centre were associated, but not student related variables).

**Table 4:** Results of step-wise regression.

			Model		
Variable	Final model terms	t-value (P-value)	r^2^	adjusted r^2^	F-value (P-value)
Number of publications	Scholarship awarded	3.43 (P = 0.001)	0.122	0.104	6.73 (P<0.001)
	Supervisor in strategic research centre	2.39 (P = 0.018)			
	Research alignment maximum score	1.79 (P = 0.076)			
Number of citations	Supervisory team maximum score	2.09 (P = 0.039)	0.072	0.059	5.70 (P = 0.004)
	Scholarship awarded	1.95 (P = 0.053)			
Number of citations per publication	Supervisor in strategic research centre	1.77 (P = 0.079)	0.062	0.049	4.86 (P = 0.009)
	Supervisory team maximum score	1.72 (P = 0.087)			
Average impact factor	Scholarship awarded	3.84 (P<0.001)	0.133	0.121	11.22 (P<0.001)
	Supervisor in strategic research centre	1.97 (P = 0.051)			

Data are t-value (P-value), r^2^, adjusted r^2^ or F-value (P-value) derived from the final step-wise regression model. See also factorial ANOVA, reported in S4 Table, which yielded similar results.

### Number of citations

On univariate analysis (Tables 2 and 3, Fig 2), primary supervisor being located in a strategic research centre (non-parametric and parametric P both≤0.010), supervisory teams who received a maximum score (both: P≤0.012), being awarded a scholarship (both: P<0.001) were associated with this outcome, but student undergraduate performance (both: P≥0.668), student research training degree outcome (e.g. first-class honours upper band; both: P≥0.237), student academic merit score (both: P≥0.080), research topic (both: P≥0.202), primary supervisor academic level (both: P≥0.482) were not.

Step-wise regression models (Table 4) showed that supervisory team who received a maximum score (P = 0.039) and the receiving a scholarship (P = 0.053), but in this case the scholarship award was not significant. Factorial ANOVA (S4 Table) yielded similar results (having a scholarship and supervisory teams who received a maximum score were associated, but not student related variables).

### Citations per publications

On univariate analysis (Tables 2 and 3, Fig 2), primary supervisor being located in a strategic research centre (non-parametric and parametric P both P≤0.009), supervisory teams who received a maximum score (non-parametric: P<0.001, parametric: P = 0.159), being awarded a scholarship (both: P≤0.048) were associated with this outcome, but student undergraduate performance (both: P≥0.640), student research training degree outcome (e.g. first-class honours upper band; both: P≥0.668), student academic merit score (both: P≥0.082), research topic (both: P≥0.185), primary supervisor academic level (both: P≥0.160) were not.

Step-wise regression models (Table 4) showed that primary supervisor being located in a strategic research centre (P = 0.079) and supervisory team achieving maximum score (P = 0.087) remained in the final model, but neither terms were significant. Factorial ANOVA (S4 Table) yielded similar results (having a scholarship and supervisory teams who received a maximum score approached, but did not reach, significance).

### Average impact factor

On univariate analysis (Tables 2 and 3, Fig 2), primary supervisor being located in a strategic research centre (non-parametric and parametric P both P≤0.001), supervisory teams who received a maximum score (both: P≤0.005), being awarded a scholarship (both: P<0.001), student academic merit score (both: P≤0.005), were associated with this outcome, but student undergraduate performance (both: P≥0.077), student research training degree outcome (e.g. first-class honours upper band; both: P≥0.238), research topic (both: P≥0.161), primary supervisor academic level (both: P≥0.125) were not.

Step-wise regression models (Table 4) showed that receiving a scholarship (P<0.001) and primary supervisor being located in a strategic research centre (P = 0.051) remained in the final model, with the latter not achieving statistical significance. Factorial ANOVA (S4 Table) yielded similar results (having a scholarship was significant, but supervisor related variables approached, but did not reach, significance; student related variables were not significant).

### Drop-out from PhD

Odds ratios for student attrition is shown in Table 1. Students were more than two times more likely to withdraw from their PhD when the supervisory team did not achieve maximum score (odds ratio [95% confidence interval] 2.88[1.39, 5.93], P = 0.004) or a scholarship was not awarded (odds ratio [95% confidence interval] 3.04[1.37, 6.73], P = 0.006). No other independent variables significantly predicted the likelihood of withdrawal.

The final multiple logistic regression model (χ^2^ = 13.80, df = 3, P = 0.003) for predicting withdrawal from PhD included maximum supervisory team score (OR = 3.29, P = 0.013; i.e. lower risk of withdrawal when the supervisor score was maximum), student undergraduate degree grades (OR = 0.58, P = 0.047; i.e. reduced risk for each GPA rank lower) and receiving a scholarship (OR = 2.30, P = 0.090; i.e. lower risk when scholarship received), albeit the latter was not significant.

### Associations between explanatory variables

Students in the highest quartile of academic merit received the most (42%) of all scholarships awarded. Of those in the highest quartile of academic merit, 79% received scholarships, compared to 62% in the second quartile, 20% in the third quartile and 22% in the lowest quartile.

Students who received a scholarship were more often supervised by strong supervisory teams (χ^**2**^ = 9.346, P = 0.002; Table 5) and by supervisors who were located in a strategic research centre (χ^**2**^ = 8.225, P = 0.004; Table 5). Supervisors who were in a strategic research centre were more likely to attract students in the highest quartile of academic merit (χ^**2**^ = 3.899, P = 0.048; Table 6). Supervisory teams who received a maximum score were more likely to attract students in the highest quartile of academic merit (χ^**2**^ = 10.147, P = 0.001; Table 6).

**Table 5:** Students who received a scholarship were most often supervised by stronger supervisory teams and supervisors who were located in a strategic research centre.

Has scholarship	Supervisor score is maximum	
	Yes	No
	**Supervisor in strategic research centre: Yes**	
**Yes**	61 (30.8)	13 (6.6)
**No**	44 (22.2)	25 (12.6)
	**Supervisor in strategic research centre: No**	
**Yes**	7 (3.5)	9 (4.5)
**No**	15 (7.6)	24 (12.1)

Data are count (percentage of total sample). See text for results from chi-squared statistics and corresponding P-values.

**Table 6:** Stronger supervisory teams and supervisors who were located in a strategic research centre were more likely to attract students in the highest quartile of academic merit.

Student is in top quartile of academic merit	Supervisor score is maximum	
	Yes	No
	**Supervisor in strategic research centre: Yes**	
**Yes**	34 (17.2)	6 (3.0)
**No**	71 (35.9)	32 (16.2)
	**Supervisor in strategic research centre: No**	
**Yes**	6 (3.0)	2 (1.0)
**No**	16 (8.1)	31 (15.7)

Data are count (percentage of total sample). See text for results from chi-squared statistics and corresponding P-values.

## Discussion

To the best of our knowledge, this is the first analysis of PhD student outcomes in relation to their research environment, their academic abilities and prior research training. The key finding was that the 'research environment', such as whether the supervisor was in a research centre or institute and the research experience of the supervision team, were most significant predictors of, with the largest effect sizes for, student outcomes. In contrast, the students' previous academic outcomes and previous research training were not predictors. Receiving a PhD scholarship had a significant influence on positive student outcomes and was more important than students being judged as having the highest academic merit. Receiving a scholarship occurred more frequently in students tied to stronger supervisory teams and supervisors in strategic research centres.

Entry to a PhD is typically restricted to those students with a minimum grade in a prior Masters or Honours degree [16]. At our university, prospective PhD students are required to have completed a research project with a dissertation of at least 25% of one year full-time study at Honours or Masters level and their grade needs to have been at least 70%. Our findings suggest that once students meet the minimum academic ability for entry into PhD, any further ability or research training above that does not influence the outcome of their PhD. This is in line with findings that scientist’s intelligence quotient does not correlate with their citation rates [17].

By contrast, it is the research environment in which the student is embedded that is decisive for the outcomes of their PhD; including the strength of their supervisory team. This is in line with the hypothesis of “accumulative advantage”, also known as “Matthew effects” in science [18] where differences between scientists at an early stage of their career become reinforced over time [19]. The standing of a PhD supervisor directly influences [8] the future career trajectory, and number of citations, their students receive throughout their career. Also, the standing of a department influences the future employment chances of its PhD graduates, on average, more than the individual achievements of those students [13]. The impact of teacher quality is seen in other areas of education [20,21], although ‘PhD supervisor quality’ is assessed differently to teacher quality in school and undergraduate education.

There are other factors known to impact the number and impact of publication outputs. Research collaboration has clearly been shown to lead to higher impact publications [22–25]. In the health-sciences field, publications of higher levels of evidence [26] are more likely to be cited. Similarly interventional (rather than observational) and prospective (rather than retrospective) studies [25,27], as well as randomised controlled trials and basic science papers [28] are more likely to be cited. Papers published in high impact factor journals will be more often cited simply for that reason [23,25]. We argue these factors are more likely to be determined by the research culture in which the student are embedded, as opposed to being determined by the student alone.

We also showed that receiving a PhD scholarship contributed to the students’ outcomes, in particular with more publications arising, more citations higher impact factor journals. In step-wise regression, we found that impact of the scholarship persisted for the number of publications and average impact factor of the journals in which the students published. This finding is in line with prior work [29] that showed PhD students receiving scholarships to support their studies published more peer reviewed papers. Similar to prior work [29], our results showed that receiving a scholarship was also associated with lower withdrawal rates.

Students were awarded scholarships based on their prior academic performance [30]. At this university, whilst the student’s academic merit contributed to 60% of their total ranking score, in practice this was the most decisive factor in determining which applicants were offered scholarships first. We show here, however, that the most significant attributes for PhD success were research environment and the performance metrics of the supervision team. How these attributes may influence employment opportunities post PhD also warrants further investigation.

Strengthening the research environment is also worthy of further investigation. Prior work [12] has shown that very few university departments rely solely on a small number of high-performing researchers for its research productivity. We show here that supervisor team quality has a key impact on the PhD student’s outcomes. Therefore, having more highly trained researchers is likely to lead to overall higher research student productivity, such as in having a higher percentage of faculty members who are at full-professor level [12]. Strategies for strengthening the research capacity of academic staff and potential supervisors include [31] structured research mentoring of academic staff, formal requirements for further academic research training.

The strengths of this analysis include being a prospective analysis of outcomes based on data that were known at the time of student selection. The limitations of the analysis were that it was focussed on one faculty at one university. It was not possible to conduct this analysis more widely at our university or at other universities as not all faculties and universities collate the same data on their PhD applicants. It would be relevant to examine such patterns at a wider range of universities, however obtaining such data from other universities is further complicated by data from scholarship ranking being confidential internal university information. Whilst this study was comprised one university, we believe its findings can easily be extrapolated to other regions of Australia and/or the world. Furthermore, we focussed on outcomes from PhDs that relate to university ranking procedures. Other outcomes, such as employment achieved post-PhD, student satisfaction, mental health are important to consider more widely.

## Conclusions

In conclusion, to best of our knowledge, our study is the first to examine the relative importance of the environment versus student ability in the allocation and outcomes of their PhD. Our key finding was that the research environment is likely more important for supporting PhD students to produce larger numbers of highly cited publications in higher impact journals. Once the minimum level of academic ability and research training is met for entry to PhD, working with a strong research focussed supervisory team, being embedded in a research intensive institute, and receiving a scholarship are also important factors for publication and citation outcomes.

## Supporting information

S1 Table Non-parametric effect sizes between the ranking criteria of the 198 unique PhD applications and researcher metrics. Data are Cohen’s d. Bold = P<0.05. GPA: Grade point average.
(DOCX)

S2 Table Parametric effect sizes between the ranking criteria of the 198 unique PhD applications and researcher metrics. Data are Cohen’s d. Bold = P<0.05. GPA: Grade point average.
(DOCX)

S3 Table Variability among variables by year of application. Dependent variables are mean (standard deviation), expect withdrawing from PhD which are number (percentage within year). Explanatory variables are number (percentage within year). GPA: Grade point average.
(DOCX)

S4 Table Results from factorial ANOVA. Data are F-value (corresponding P-value). ANOVA fits explanatory variables sequentially to the dependent variables. Explanatory variables were fitted to the dependent variables in the order above (i.e. top variable at left fitted first, followed by the second to top variable). This therefore accounted for potential association of student related factors first to PhD outcomes, with then having a scholarship and then supervisor related factors considered. Despite accounting for student related variables first, having a scholarship and supervisor quality were most consistently associated with outcomes from a student’s PhD.
(DOCX)
