# Supplementary material for: Do successful PhD outcomes reflect the research environment rather than academic ability?
Source: PLoS One. 2020 Aug 5;15(8):e0236327. doi: 10.1371/journal.pone.0236327 (PMC7406039; doi:10.1371/journal.pone.0236327)
Supplement: S1 Table — Data are Cohen’s d. Bold = P<0.05. GPA: Grade point average. (DOCX) [file pone.0236327.s001.docx]

**S1 Table.** Non-parametric effect sizes between the ranking criteria of the 198 unique PhD applications and researcher metrics.

| Variable | Number of publications | Number of citations | Number of citations per publication | Average impact factor |
| --- | --- | --- | --- | --- |
| Student research training degree | 0.149 | 0.146 | 0.180 | 0.166 |
| 1^st^ class honours (top) vs 1^st^ class honours (middle) | 0.077 | 0.042 | 0.035 | 0.110 |
| 1^st^ class honours (top) vs 1^st^ class honours (lower) | 0.223 | 0.229 | 0.190 | 0.167 |
| 1^st^ class honours (top) vs 2^nd^ class honours | 0.319 | 0.180 | 0.142 | **0.364** |
| 1^st^ class honours (middle) vs 1^st^ class honours (lower) | 0.181 | 0.230 | 0.310 | 0.102 |
| 1^st^ class honours (middle) vs 2^nd^ class honours | 0.322 | 0.157 | 0.113 | 0.300 |
| 1^st^ class honours (lower) vs 2^nd^ class honours | 0.140 | 0.020 | 0.023 | 0.169 |
| Student undergraduate rank | 0.163 | 0.189 | 0.181 | 0.066 |
| GPA≥80% plus prizes vs GPA≥80% | 0.240 | 0.258 | 0.258 | 0.290 |
| GPA≥80% plus prizes vs GPA≥70% and <80% | 0.048 | 0.105 | 0.197 | 0.252 |
| GPA≥80% plus prizes vs GPA≥60% and <70% | 0.359 | 0.329 | 0.385 | 0.542 |
| GPA≥80% vs GPA≥70% and <80% | 0.131 | 0.126 | 0.060 | 0.054 |
| GPA≥80% vs GPA≥60% and <70% | 0.070 | 0.072 | 0.079 | 0.171 |
| GPA≥70% and <80% vs GPA≥60% and <70% | 0.214 | 0.148 | 0.129 | 0.175 |
| Student had prior publication (yes vs no) | 0.116 | 0.090 | 0.115 | 0.140 |
| Student academic merit | **0.394** | 0.281 | 0.279 | **0.532** |
| 1^st^ vs 2^nd^ quartile | **0.467** | 0.341 | 0.370 | **0.632** |
| 1^st^ vs 3^rd^ quartile | **0.426** | 0.305 | 0.269 | **0.586** |
| 1^st^ vs 4^th^ quartile | **0.654** | **0.539** | **0.535** | **0.766** |
| 2^nd^ vs 3^rd^ quartile | 0.062 | 0.044 | 0.072 | 0.066 |
| 2^nd^ vs 4^th^ quartile | 0.127 | 0.165 | 0.140 | 0.129 |
| 3^rd^ vs 4^th^ quartile | 0.182 | 0.187 | 0.200 | 0.201 |
| Supervisor in institute or research centre (yes vs no) | **0.535** | **0.549** | **0.528** | **0.621** |
| Supervisor academic level at application | 0.235 | 0.110 | 0.125 | 0.220 |
| Full-professor vs associate professor | 0.161 | 0.034 | 0.001 | 0.045 |
| Full-professor vs senior lecturer or lecturer | **0.355** | 0.194 | 0.172 | 0.290 |
| Associate professor vs senior lecturer or lecturer | 0.232 | 0.158 | 0.163 | 0.317 |
| Supervisory team achieved maximum score (yes vs no) | **0.604** | **0.517** | **0.540** | **0.578** |
| Alignment of research achieved maximum score (yes vs no) | 0.049 | 0.050 | 0.125 | 0.141 |
| Scholarship awarded (yes vs no) | **0.951** | **0.814** | **0.738** | **0.887** |

Data are Cohen’s d. Bold = P<0.05. GPA: Grade point average.
