# Supplementary material for: Do successful PhD outcomes reflect the research environment rather than academic ability?
Source: PLoS One. 2020 Aug 5;15(8):e0236327. doi: 10.1371/journal.pone.0236327 (PMC7406039; doi:10.1371/journal.pone.0236327)
Supplement: S2 Table — Data are Cohen’s d. Bold = P<0.05. GPA: Grade point average. (DOCX) [file pone.0236327.s002.docx]

**S2 Table.** Parametric effect sizes between the ranking criteria of the 198 unique PhD applications and researcher metrics.

| Variable | Number of publications | Number of citations | Number of citations per publication | Average impact factor |
| --- | --- | --- | --- | --- |
| Student research training degree | 0.320 | 0.478 | 0.208 | 0.668 |
| 1^st^ class honours (top) vs 1^st^ class honours (middle) | 0.164 | 0.123 | 0.020 | 0.231 |
| 1^st^ class honours (top) vs 1^st^ class honours (lower) | 0.362 | 0.221 | 0.111 | 0.169 |
| 1^st^ class honours (top) vs 2^nd^ class honours | 0.000 | 0.273 | 0.243 | 0.346 |
| 1^st^ class honours (middle) vs 1^st^ class honours (lower) | 0.260 | 0.146 | 0.081 | 0.081 |
| 1^st^ class honours (middle) vs 2^nd^ class honours | 0.117 | 0.314 | 0.210 | 0.209 |
| 1^st^ class honours (lower) vs 2^nd^ class honours | 0.267 | 0.347 | 0.066 | 0.256 |
| Student undergraduate rank | 0.234 | 0.065 | 0.334 | 0.810 |
| GPA≥80% plus prizes vs GPA≥80% | 0.213 | 0.076 | 0.204 | 0.581 |
| GPA≥80% plus prizes vs GPA≥70% and <80% | 0.072 | 0.029 | 0.407 | 0.550 |
| GPA≥80% plus prizes vs GPA≥60% and <70% | 0.058 | 0.000 | 0.121 | 0.588 |
| GPA≥80% vs GPA≥70% and <80% | 0.240 | 0.032 | 0.134 | 0.097 |
| GPA≥80% vs GPA≥60% and <70% | 0.106 | 0.060 | 0.054 | 0.140 |
| GPA≥70% and <80% vs GPA≥60% and <70% | 0.124 | 0.022 | 0.186 | 0.215 |
| Student had prior publication (yes vs no) | 0.171 | 0.023 | 0.043 | 0.158 |
| Student academic merit | 0.220 | 0.099 | 0.208 | **0.628** |
| 1^st^ vs 2^nd^ quartile | 0.110 | 0.068 | 0.193 | **0.451** |
| 1^st^ vs 3^rd^ quartile | 0.117 | 0.046 | 0.096 | **0.525** |
| 1^st^ vs 4^th^ quartile | 0.231 | 0.058 | 0.029 | **0.549** |
| 2^nd^ vs 3^rd^ quartile | 0.000 | 0.097 | 0.227 | 0.057 |
| 2^nd^ vs 4^th^ quartile | 0.107 | 0.000 | 0.083 | 0.112 |
| 3^rd^ vs 4^th^ quartile | 0.111 | 0.086 | 0.093 | 0.073 |
| Supervisor in institute or research centre (yes vs no) | **0.497** | **0.413** | **0.423** | **0.534** |
| Supervisor academic level at application | 0.325 | 0.040 | 0.332 | 0.340 |
| Full-professor vs associate professor | 0.256 | 0.000 | 0.228 | 0.230 |
| Full-professor vs senior lecturer or lecturer | 0.307 | 0.040 | 0.096 | 0.115 |
| Associate professor vs senior lecturer or lecturer | 0.060 | 0.036 | 0.301 | 0.309 |
| Supervisory team achieved maximum score (yes vs no) | **0.368** | **0.376** | 0.211 | **0.424** |
| Alignment of research achieved maximum score (yes vs no) | 0.034 | 0.249 | 0.124 | 0.187 |
| Scholarship awarded (yes vs no) | **0.621** | **0.474** | **0.286** | **0.729** |

Data are Cohen’s d. Bold = P<0.05. GPA: Grade point average.
