# Supplementary material for: Do successful PhD outcomes reflect the research environment rather than academic ability?
Source: PLoS One. 2020 Aug 5;15(8):e0236327. doi: 10.1371/journal.pone.0236327 (PMC7406039; doi:10.1371/journal.pone.0236327)
Supplement: S3 Table — Dependent variables are mean (standard deviation), expect withdrawing from PhD which are number (percentage within year). Explanatory variables are number (percentage within year). GPA: Grade point average. (DOCX) [file pone.0236327.s003.docx]

**S3 Table.** Variability among variables by year of application.

| Variable | Year of application | | | | P-value |
| --- | --- | --- | --- | --- | --- |
|  | **2011** | **2012** | **2013** | **2014** |  |
| ***Dependent variables*** | | | | | |
| Number of publications | 2.3 (3.6) | 3.1 (4.9) | 3.5 (4.7) | 2.8 (4.7) | P=0.549 |
| Number of citations | 17.4 (33.6) | 27.9 (66.5) | 18.3 (29.8) | 15.3 (46.5) | P=0.451 |
| Number of citations per publication | 3.6 (5.1) | 4.9 (10.9) | 3.0 (4.5) | 2.9 (5.1) | P=0.380 |
| Average impact factor | 1.2 (1.6) | 1.4 (2.0) | 2.3 (3.4) | 1.7 (2.0) | P=0.067 |
| Withdrawing from PhD |  |  |  |  |  |
| Yes | 9 (15.0) | 7 (14.6) | 10 (26.3) | 11 (21.2) | P=0.435 |
| ***Explanatory variables*** | | | | | |
| Student research training degree |  |  |  |  |  |
| 1st class honours, top | 23 (38.3) | 22 (50.0) | 20 (55.6) | 24 (54.4) | P=0.539 |
| 1st class honours, middle | 16 (28.1) | 5 (11.4) | 7 (19.4) | 6 (13.0) | P=0.088 |
| 1st class honours, lower | 6 (10.5) | 9 (20.5) | 1 (2.8) | 8 (17.4) | P=0.113 |
| 2nd class honours | 12 (21.1) | 8 (18.2) | 8 (22.2) | 7 (15.2) | P=0.756 |
| Student undergraduate rank |  |  |  |  |  |
| GPA≥80% plus prizes | 2 (3.6) | 5 (13.9) | 4 (13.8) | 3 (6.4) | P=0.404 |
| GPA≥80% | 27 (49.1) | 7 (19.4) | 4 (13.8) | 8 (17.0) | **P<0.001** |
| GPA≥70% and <80% | 21 (38.2) | 19 (52.8) | 19 (65.5) | 20 (42.6) | P=0.519 |
| GPA≥60% and <70% | 5 (9.1) | 5 (13.9) | 2 (6.9) | 16 (34.0) | **P=0.001** |
| Student had prior publication |  |  |  |  |  |
| Yes | 9 (15.0) | 7 (14.6) | 10 (26.3) | 11 (21.2) | P=0.435 |
| Student academic merit |  |  |  |  |  |
| 1st quartile | 17 (28.3) | 7 (14.6) | 12 (31.6) | 12 (23.1) | P=0.247 |
| 2nd quartile | 11 (18.3) | 17 (35.4) | 11 (29.0) | 11 (21.2) | P=0.180 |
| 3rd quartile | 12 (20.0) | 18 (37.5) | 6 (15.8) | 14 (26.9) | P=0.087 |
| 4th quartile | 20 (33.3) | 6 (12.5) | 9 (23.7) | 15 (28.9) | P=0.085 |
| Supervisor in institute or research centre |  |  |  |  |  |
| Yes | 37 (61.7) | 34 (70.8) | 29 (76.3) | 43 (82.7) | P=0.088 |
| Supervisor academic level at application |  |  |  |  |  |
| Full-professor | 12 (20.0) | 15 (31.3) | 10 (26.3) | 16 (30.8) | P=0.508 |
| Associate professor | 13 (21.7) | 12 (25.0) | 13 (34.2) | 14 (26.9) | P=0.584 |
| Senior lecturer or lecturer | 23 (38.3) | 20 (41.7) | 15 (39.5) | 21 (40.4) | P=0.988 |
| Supervisory team achieved maximum score |  |  |  |  |  |
| Yes | 42 (70.0) | 31 (64.6) | 26 (68.4) | 28 (53.9) | P=0.308 |
| Alignment of research achieved maximum score |  |  |  |  |  |
| Yes | 55 (93.2) | 37 (77.1) | 31 (81.6) | 42 (80.8) | P=0.115 |
| Scholarship awarded |  |  |  |  |  |
| Yes | 22 (36.7) | 24 (50.0) | 16 (42.1) | 30 (57.7) | P=0.183 |

Dependent variables are mean (standard deviation), expect withdrawing from PhD which are number (percentage within year). Explanatory variables are number (percentage within year). GPA: Grade point average.
