# Supplementary material for: Do successful PhD outcomes reflect the research environment rather than academic ability?
Source: PLoS One. 2020 Aug 5;15(8):e0236327. doi: 10.1371/journal.pone.0236327 (PMC7406039; doi:10.1371/journal.pone.0236327)
Supplement: S4 Table — Data are F-value (corresponding P-value). ANOVA fits explanatory variables sequentially to the dependent variables. Explanatory variables were fitted to the dependent variables in the order above (i.e. top variable at left fitted first, followed by the second to top variable). This therefore accounted for potential association of student related factors first to PhD outcomes, with then having a scholarship and then supervisor related factors considered. Despite accounting for student related variables first, having a scholarship and supervisor quality were most consistently associated with outcomes from a student’s PhD. (DOCX) [file pone.0236327.s004.docx]

**S4 Table. Results from factorial ANOVA.**

|  | **Number of publications** | **Number of citations** | **Number of citations per publication** | **Average impact factor** |
| --- | --- | --- | --- | --- |
| Student research training degree | 0.8 (P=0.583) | 0.8 (P=0.517) | 1.2 (P=0.293) | 1.2 (P=0.294) |
| Student undergraduate rank | 0.8 (P=0.481) | 0.1 (P=0.938) | 0.5 (P=0.687) | 1.9 (P=0.131) |
| Student academic merit | 2.4 (P=0.068) | 0.7 (P=0.532) | 0.5 (P=0.698) | 2.0 (P=0.111) |
| Student had prior publication | 0.2 (P=0.628) | 0.2 (P=0.698) | 0.0 (P=0.918) | 0.2 (P=0.641) |
| Alignment of research achieved maximum score | 0.0 (P=0.962) | 0.7 (P=0.495) | 1.5 (P=0.230) | 0.4 (P=0.669) |
| Scholarship awarded | **13.4 (P<0.001)** | **6.7 (P=0.010)** | 3.4 (P=0.067) | **10.6 (P=0.001)** |
| Supervisory team achieved maximum score | **4.0 (P=0.047)** | **4.5 (P=0.035)** | 3.5 (P=0.064) | 3.3 (P=0.070) |
| Supervisor in institute or research centre | **4.1 (P=0.045)** | 1.7 (P=0.189) | 2.2 (P=0.142) | 3.2 (P=0.075) |
| Supervisor academic level at application | 0.7 (P=0.533) | 0.4 (P=0.779) | 0.7 (P=0.579) | 0.1 (P=0.972) |

Data are F-value (corresponding P-value). ANOVA fits explanatory variables sequentially to the dependent variables. Explanatory variables were fitted to the dependent variables in the order above (i.e. top variable at left fitted first, followed by the second to top variable).This therefore accounted for potential association of student related factors first to PhD outcomes, with then having a scholarship and then supervisor related factors considered. Despite accounting for student related variables first, having a scholarship and supervisor quality were most consistently associated with outcomes from a student’s PhD.
