# Supplementary material for: Do successful PhD outcomes reflect the research environment rather than academic ability?
Source: PLoS One. 2020 Aug 5;15(8):e0236327. doi: 10.1371/journal.pone.0236327 (PMC7406039; doi:10.1371/journal.pone.0236327)
Supplement: Attachment — Submitted filename: 2019_12_05_PONE-D-19-27758_Response.docx [file pone.0236327.s005.docx]

1. Is the manuscript technically sound, and do the data support the conclusions?

**Reviewer #1:** No

**Reviewer #2:** Yes

**Author response:** Reviewers differ in their opinions.

We thank Reviewer #2 for remarking this was *‘a great piece of work’*. We also thank both Reviewer #1 and #2 for provided useful ideas. We have responded to the comments of both reviewers and updated the manuscript according. We believe the manuscript to be technically sound and that the data support the conclusions. Notably, as often happens with statistics, especially in larger sample sizes such as here, the different analysis approach did not change the main findings. We believe this contributes to the robustness of the manuscript.

2. Has the statistical analysis been performed appropriately and rigorously?

**Reviewer #1:** No

**Reviewer #2:** Yes

**Author response:** Reviewers differ in their opinions.

We consulted with a professorial biostatistician for the statistical approach during the analysis of the data whilst drafting the original manuscript.

Reviewer #1 may have missed that we included non-parametric statistics in our analyses. We agree there are concerns using only parametric stats (e.g. assumptions of normality). Therefore, on statistical advice, we included non-parametric statistics. The results of these analyses were essentially the same as those from the parametric stats. This often is the case with larger sample sizes (see Central Limit Theorem).

Reviewer #2 considered the statistics appropriate in revising the manuscript. We further consulted with the same professorial biostatistician and are confident the statistics are appropriate

We respond to more specific comments on this point below and have revised the manuscript, accordingly, adding further data Tables and Supplemental Data.

3. Have the authors made all data underlying the findings in their manuscript fully available?

**Reviewer #1:** No

**Reviewer #2:** Yes

**Author response:** Reviewers differ in their opinions.

Our reasons for being unable to provide raw data on the internet were disclosed with the submission. Some of the data are internal confidential university data which could potentially re-identify individual students included in the analyses. The data can be accessed by others with an ethically conform request.

4. Is the manuscript presented in an intelligible fashion and written in standard English?

**Reviewer #1:** Yes

**Reviewer #2:** Yes

**Author response:** No response required

**Reviewer #1**

**Reviewer #1:** The present paper covers an important topic, i.e. research policy and selection of PhD students. Very little research has been done on the association between student characteristics, the scientific environment, research output and benefits for society. In this context, the paper covers an important topic and the researchers may have had access to some interesting data.

**Author response:** Thank-you for pointing out the novelty and importance of our work.

**Reviewer #1:** The study is based on data from one Australian university. Of the 324 applications submitted to the university, 198 students were enrolled in the PhD program, 120 completed the program, 31 are still enrolled, 37 withdraw after starting, and 11 withdrew before starting.

**Author response:** The reviewer reiterates the data sample. No response required. Responses to the reviewer comments based on this summary are given below.

**Reviewer #1:** Unfortunately, very few conclusions can be drawn from this small study due to several issues with the statistical analysis and study design.

**Author response:** The data are unique and hard to get at wide scale: (a) key aspects of these data are derived from internal university (i.e. confidential) scholarship ranking panels, (b) universities are typically reluctant to release such information and are highly unlikely to share such data with external investigators due to strict privacy laws, (c) collection of the information is inconsistent between institutions, (d) even within our own university, other Faculties do not collect similar information. Thus, the data are unique.

The ~200 students in the sample are, in light of the granularity and depth of the data, a large sample size. Data involving 1000s of students is difficult to obtain and impossible to obtain at the level of granularity that we have analysed.

We believe our work is the first on this important topic and the difficulty in attaining the data highlights the uniqueness of our manuscript.

**Reviewer #1:** First of all, the study is a cross-sectional study, and not all students have the same follow-up time. For example, 31 students are still enrolled in the program at the time of the data analysis. Since the inclusion period is from 2010-2013, some students may have been followed for 8 years, others for 4-5 years (please see Rothman KJ. Epidemiology. An Introduction. P. 97. Oxford University Press, 2nd ed., 2012). Moreover, the reader cannot really evaluate the underlying distribution of the data and their validity.

**Author response:** We disagree with the Reviewer’s assertion that this is a cross-sectional study. A cross-sectional study is a comparison at a single time-point. In the current study, we retrospectively analysed an entire cohort over 4+ years of follow-up. A cross-sectional study is where all participants are examined at the same time with no follow-up data.

We painstakingly and carefully combined different data sets, which included checking other internal university databases for whether students changed their name during candidature (as they might publish manuscripts under their new surname, such as after getting married), to ensure we did not miss any one in this cohort.

RE: follow-up period. we thank the reviewer for this comment and agree that some students will have had a longer time to amass publications and citations. We conducted further analyses and student quality and other examined factors did not vary from one year to the next. We have presented this in Supplementary Table 3.

The parameters that were related to outcomes from PhD were stable over the years considered. Only student undergraduate rank varies over the years depending on year of application. Yet this parameter was unrelated to PhD outcomes. Thus, the longer follow-up period for applicants from 2011 versus those in later years will not impact the findings.

**Reviewer #1:** To mention some examples: The mean number of publications was 2.8 with a standard deviation of 4.4; the average impact factor was 1.9 with a standard deviation of 2.36; the mean number of citations per publication was 3.5 with a standard deviation of 7.4; and the total citation was 19.6 with a standard deviation of 49.8. 95% of a normal distribution is to be calculated as mean 1.96 ± standard deviation. This means that if the data were to be correctly described large proportion of students would have negative number of publications, negative impact factor, negative citations, and negative total citations. This does not make sense.

**Author response:** We thank the reviewer for this comment. We identified concerns with using only parametric stats (e.g. assumptions of normality). Therefore, after consulting with a professorial biostatistician prior to the original submission, we included non-parametric statistics. The results of these analyses were essentially the same as those from the parametric stats.

We have added a separate table for the non-parametric statistics (presenting medians and interquartile ranges; new Table 2) and retain the original table showing means and standard deviations (Table 3). As stated in the original manuscript, the results are largely the same. This is typical of larger datasets where parametric and non-parametric analyses give largely the same main findings (see Central Limit Theorem).

Notably, we consulted with the same professorial biostatistician for the original submission and this revision.

**Reviewer #1:** Moreover, it is not clear if this is an etiologic or prediction study (please see Clayton D, Hills M. Statistical Models in Epidemiology, P. 271, Chapter 27, Choice and Interpretation of Models. Oxford University Press, 1993). If it is an etiologic study, a step-wise logistic regression model does not make much sense (please see Rothman KJ. Epidemiology. An Introduction. P. 194. Oxford University Press, 2nd ed., 2012).

**Author response:** We sought to examine what baseline (at scholarship ranking) factors were associated with more/less publications, more/less citations and higher/lower impact factor. This is important for choosing (potentially: ‘*predicting*’) which students are more likely to have the best outcome.

Our work would then fall under the reviewer’s ‘prediction’ category, making the criticism of step-wise logistic regression not applicable.

We consulted with a professorial biostatistician for the original submission and this revision, hence we are confident that these statistics have been conducted appropriately.

**Reviewer #1:** This relatively small study has limited statistical precision of the estimates as evident from Table 2.

**Author response:** As mentioned prior:

*The data are unique and hard to get at wide scale: (a) key aspects of these data are derived from internal university (i.e. confidential) scholarship ranking panels, (b) universities are typically reluctant to release such information and are highly unlikely to share such data with external investigators due to strict privacy laws, (c) collection of the information is inconsistent between institutions, (d) even within our own university, other Faculties do not collect similar information. Thus, the data are unique.*

*The ~200 students in the sample are, in light of the granularity and depth of the data, a large sample size. Data involving 1000s of students is difficult to obtain and impossible to obtain at the level of granularity that we have analysed.*

*We believe our work is the first on this important topic and the difficulty in attaining the data highlights the uniqueness of our manuscript.*

**Reviewer #1:** Only significant results have been marked with bold, and many strong associations are ignored simply because they are not statistically significant (please see Amrhein V et al. Scientists rise up against statistical significance. Nature. 2019;567:305-307).

**Author response:** In the revised manuscript, we presented effect sizes (see new Figure 2 and Supplemental Tables 1 and 2). As often happens in statistical analyses, the statistical significance is associated with the largest effect sizes.

Aside from statistically significant effects, we also included in the Discussion (paragraph 1) consideration of effect sizes.

Notably, we consulted with a professorial biostatistician for the original submission and this revision.

**Reviewer #1:** Moreover, the table also shows that the data cannot be described with standard deviation.

**Author response:** As mentioned prior:

*We have added a separate table for the non-parametric statistics (presenting medians and interquartile ranges; new Table 2) and retain the original table showing means and standard deviations (Table 3). As stated in the original manuscript, the results are largely the same. This is typical of larger datasets where parametric and non-parametric analyses give largely the same main findings (see Central Limit Theorem).*

**Reviewer #1:** Another problem with logistic regression is the rare outcome assumption, which seem to be the case in the present paper (please see Hosmer DW, Lemeshow S, Sturdivant RX. Applied logistic regression, 3rd ed. P. 51. Hoboken: Wiley, 2013).

**Author response:** This comment refers to the drop-out of students from PhD.

In our sample 19.7% of students dropped out of PhD at the Government’s census cut-off date. We posit that 20% is a frequent event rather than a ‘rare outcome’. However, this same topic also refers to the sample size and potential for bias in the logit coefficients (King & Zeng "Logistic Regression in Rare Events Data". Political Analysis, 2001. 9: 137-163; <https://gking.harvard.edu/files/0s.pdf>). We take on this consideration.

In the revised manuscript we implement the Penalized Maximum Likelihood Estimation proposed by Firth ("Bias reduction of maximum likelihood estimates" Biometrika 80:27-38; <https://doi.org/10.1093/biomet/80.1.27>). This is considered the best approach to implementing logistic regression in sample sizes such as in the current study.

The findings of the logistic regression analyses are similar to those presented in the original manuscript.

**Reviewer #1:** In addition to lack of statistical power, this type of non-randomized observational study should not focus on statistical significance, but on estimation of the effect (please see Rothman KJ. Six persistent research misconceptions. J Gen Intern Med. 2014;29:1060-4).

**Author response:** As mentioned prior:

RE: Sample size:

*The data are unique and hard to get at wide scale: (a) key aspects of these data are derived from internal university (i.e. confidential) scholarship ranking panels, (b) universities are typically reluctant to release such information and are highly unlikely to share such data with external investigators due to strict privacy laws, (c) collection of the information is inconsistent between institutions, (d) even within our own university, other Faculties do not collect similar information. Thus, the data are unique.*

*The ~200 students in the sample are, in light of the granularity and depth of the data, a large sample size. Data involving 1000s of students is difficult to obtain and impossible to obtain at the level of granularity that we have analysed.*

*We believe our work is the first on this important topic and the difficulty in attaining the data highlights the uniqueness of our manuscript.*

RE: effect sizes:

*In the revised manuscript, we presented effect sizes (see new Figure 2 and Supplemental Tables 1 and 2). As often happens in statistical analyses, the statistical significance is associated with the largest effect sizes.*

*Aside from statistically significant effects, we also included in the Discussion (paragraph 1) consideration of effect sizes.*

*Notably, we consulted with a professorial biostatistician for the original submission and this revision.*

**Reviewer #1:** The study is conducted at a university in Australia as mentioned by the authors, but the external validity should be discussed in more detail.

**Author response:** As mentioned prior:

RE: Sample size:

*The data are unique and hard to get at wide scale: (a) key aspects of these data are derived from internal university (i.e. confidential) scholarship ranking panels, (b) universities are typically reluctant to release such information and are highly unlikely to share such data with external investigators due to strict privacy laws, (c) collection of the information is inconsistent between institutions, (d) even within our own university, other Faculties do not collect similar information. Thus, the data are unique.*

*The ~200 students in the sample are, in light of the granularity and depth of the data, a large sample size. Data involving 1000s of students is difficult to obtain and impossible to obtain at the level of granularity that we have analysed.*

*We believe our work is the first on this important topic and the difficulty in attaining the data highlights the uniqueness of our manuscript.*

Furthermore, we discuss this further in the Discussion (2^nd^ last paragraph).

Notably, Reviewer #2 stated *‘Although this is a regionalized study, it can easily extrapolate its findings to other areas of the globe’*.

**Reviewer #1:** Overall, the paper covers an important topic, but the study design and the statistical analysis as well as the small sample size make it impossible to draw any valid conclusions.

**Author response:** We agree this is an important topic. We think the reviewer had some very good points. In light of the Reviewer’s comments, we have revised the manuscript and believe that it is much stronger as a consequence.

**Reviewer #2**

**Reviewer #2:** The manuscript that aims to analyzes the PhD students performance based on their previous academic achievement, research environment or supervisor importance is a great piece of work. Basically, the article puts numbers in parameters that the whole scientific community already has an idea, even qualitatively.

**Author response:** Thank-you for your positive comments on our manuscript.

**Reviewer #2:** Although this is a regionalized study, it can easily extrapolate its findings to other areas of the globe, as the data discussed are important in worldwide PhD programs.

**Author response:** We agree with the comment. We discuss this further in the Discussion (2^nd^ last paragraph).

**Reviewer #2:** The only suggestion, which may or may not be accepted, would be the inclusion of some graphs, at least for the most significant findings, since understanding the tables is hampered by the number of presented data.

**Author response:** Thank-you for this suggestion. In the revised manuscript we include a new Figure 2 which displays the effect sizes of the main findings.
