# Supplementary material for: Do successful PhD outcomes reflect the research environment rather than academic ability?
Source: PLoS One. 2020 Aug 5;15(8):e0236327. doi: 10.1371/journal.pone.0236327 (PMC7406039; doi:10.1371/journal.pone.0236327)
Supplement: Attachment — Submitted filename: 2020_05_19_PONE-D-19-27758R2_Response.docx [file pone.0236327.s006.docx]

**Reviewer #1**

Reviewer #1: Thank you very much for giving me the opportunity to review the revised paper and the authors’ response. The study focuses on an important topic, but the authors have only responded to the reviewer comments to a limited extent. Their main reply is that their data are unique and that they have consulted a statistician. However, the manuscript still has several issues.

**Author response:** Thank-you for re-reviewing our manuscript. Your initial comments, as well as those below, have strengthened the manuscript.

The material consists of fewer than 200 persons and the authors have conducted several cross-sectional analyses that the authors did not critically consider.

**Author response:** Respectfully, we responded to this in detail in our prior Response and updated the manuscript accordingly. Please refer to our prior response.

There is a large number of p-values in the manuscript yet the authors did not give any thought to statistical power in this small study population.

**Author response:** Respectfully, we responded to this in detail in our prior Response and updated the manuscript accordingly.

Moreover, the world’s leading and prominent scientists warned against this type of analysis, that is, statistical significance testing, in which only significant results are highligted. Please see Nature: https://www.nature.com/articles/d41586-019-00857-9

**Author response:** We responded to this in detail in our prior Response and updated the manuscript accordingly. Please refer to our prior response.

For this study to be a cohort analysis, the persons should either have had the same follow-up time, or the authors should have taken the various follow-up periods into consideration in the statistical analysis. This is not the case. The present analysis is solely cross-sectional.

**Author response:** We responded to this in detail in our prior Response and updated the manuscript accordingly.

It seems unclear to the authors if they have conducted a prediction study, or a causal study. It seems as if they tend to believe that their study is more of a causal study than a prediction study.

**Author response:** We responded to this in detail in our prior Response and updated the manuscript accordingly.

It is furthermore not clear what the authors want to estimate by use of odds ratios – is it prevalence rate ratio, incidence rate ratio, or relative risks? The study design does not meet the criteria for the latter two.

**Author response:** We have reviewed the manuscript to ensure that ‘odds ratio’, rather than the abbreviation ‘OR’, is used throughout to reduce confusion.

Odds ratios are a form of effect size that measure the association between and exposure and an outcome. They represent the odds that an outcome will occur given a particular exposure, compared to the odds of occurring in absence of said exposure. For further information, please see: <https://www.ncbi.nlm.nih.gov/pmc/articles/PMC2938757/>

Risk ratio, IRR are different measures. Please refer to this manuscript for further information <https://www.sciencedirect.com/science/article/pii/S0085253815527464>

It still does not make any sense to report variables by mean and standard deviation.

**Author response:** Respectfully, we responded to this in detail in our prior Response and updated the manuscript accordingly. Please refer to our prior response.

**Reviewer #2**

**Reviewer #2**: Since the first submission, I found the manuscript interesting. Few studies, even if regionalized like this, show so clearly the importance of the group in which the student is inserted. However, the title may sound aggressive in the way it is written. My opinion is that the words could be less impactful in the title...even if the results clearly show the importance of the group in the researcher formation

**Author response:** Thank-you for re-reviewing our manuscript. We have updated the title to make it a question and thus make weaker claims: “*Do successful PhD outcomes reflect the research environment rather than academic ability?”*.

We have also updated other sections of the manuscript to make weaker claims and add the use of qualifiers.

**Reviewer #3**

**Reviewer #3**: The manuscript studies factors related to PhD outcome. This is an important topic. However, my primary concern is that the manuscript has made strong claims that were not supported by the analysis.

**Author response:** Thank-you for taking the time to review our manuscript and provide your input. This has led to a stronger manuscript. We respond to your specific comments below.

The title and conclusions made in the manuscript, including adding the use of qualifiers, have been altered to reduce the strength of the claims made. E.g. the title is now posed as a question.

**Reviewer #3**: For starters, in the economics of education literature, there are essentially two schools of thought: (1) education signals how good a student is, irrespective of the quality of the training; and (2) education provides training. It is important to disentangle the two effects, and there have been a lot of studies trying to do this. Therefore, I am not convinced that “To the best of our knowledge, this is the first analysis of PhD student outcomes in relation to their research environment, their academic abilities and prior research training.” The authors may want to do more literature search on this topic.

**Author response:** We agree that there may be data from other fields of research (e.g. into undergraduate outcomes) that relate to this issue.

The main challenge comparing data from research into other areas of education is that there is often a high bar for student entry to PhD programs (e.g. prior post-graduate research training, minimum scores from this training). Thus, there are inherent limitations that impair comparing these ‘high achievers’ to other areas. We highlight this in the Abstract, in the Introduction and in the Discussion.

We have included data from such areas of work to expand the Discussion section of the manuscript: “*The impact of teacher quality is seen in other areas of education 20,21, although ‘PhD supervisor quality’ is assessed differently to teacher quality in school and undergraduate education.*” Based on a repeated in depth search of the literature, we still maintain this is the first analysis of PhD student outcomes that examines research environment, their academic abilities and prior research training within the same collective sample. Thus we have not altered this claim. The claims we make overall in terms of the outcome of that analysis have been softened.

If the reviewer is suggesting that the prior research training either signals how ‘ready’ the student is for PhD studies versus how ‘good’ the student is then we cannot comment on this. We cannot examine the ‘quality’ of their prior research training, rather their ‘score’ from this prior training. Most of the PhD applicants come from our internal university programs, so the ‘quality’ of research training provided will largely be the same for most PhD applicants.

**Reviewer #3**: A related comment is that, in the paper’s context, the fact that some students are able to do research in a strategic research center and their supervisory teams who got maximum scores may already signal that they may have better academic abilities, thus have better outcomes, as observed in the paper. In other words, it is academic abilities that affect PhD outcome.

**Author response:** Thank-you for this comment. The multivariate models assess this: by having scholarship awarded, supervisor variables and student variables in the model, and seeing which explanatory variables remain in the final model/significant, this tells us which are more important. These analyses showed that supervisor, not student, variables remained. Also important was receiving a scholarship.

Nonetheless, in the updated manuscript we have included further analysis of the association between student academic merit and supervisors being in an SRC and supervisor score (Table 6). In the Results section, we add: “*Supervisors who were in a strategic research centre were more likely to attract students in the highest quartile of academic merit (χ²=3.899, P=0.048; Table 6). Supervisory teams who received a maximum score were more likely to attract students in the highest quartile of academic merit (χ²=10.147, P=0.001; Table 6).*” Again, by themselves these numbers are not highly informative on the question you pose in your comment as they are univariate associations.

**Reviewer #3**: It remains unclear to me what the operational definitions of “research environment” and “academic ability” are. Which variables fall into which category? Does scholarship reflect academic ability? If so, the observation that students who are awarded scholarships have more papers/citations and are less likely to withdraw from PhD directly refuted the major claim of the paper.

**Author response:** Thank-you for pointing this out. Figure 2 showed the categorisation and in the updated statistical analyses section we now explicitly state which category which variable belongs to.

RE: Does scholarship reflect academic ability?

The multivariate models assessed this: by having scholarship awarded, supervisor variables and student variables in the model, and seeing which explanatory variables remain in the final model/significant, this tells us which are more important. These analyses showed that supervisor, not student, variables remained. Also important was receiving a scholarship. Overall these analyses suggest that students academic ability, once they have passed the minimum threshold of requirements for PhD entry, are not related to the outcomes of their PhD.

**Reviewer #3**: There are two variables that indicate whether strategic alignment score and supervisor team score achieve maximum. Why focusing on maximum? Wouldn’t a maximum score emphasize the very best?

**Author response:** We state in the updated Methods section: “*The presence or absence of a maximum score was taken for this variable as there were few instances of low scores on this criterion and data were skewed to the maximum score*”

E.g. 83.3% of applications had the maximum score for alignment (Table 1) and 64.1% maximum score for supervisor team.

Here we show the distribution of the supervisor team scores (left) and alignment scores (right).

|  |  |
| --- | --- |

On the advice of a statistician, we treated these variables as categorical (maximum score versus not-maximum score)

**Reviewer #3**: The current of flow in the results section is quite confusing, alternating between different variables and outcomes. I’d suggest focusing on one outcome at a time and for each outcome describing univariate analysis and regression analysis. A summary table that indicates the associations of each independent variable and each outcome is also helpful.

**Author response:** Thank you, we have restructured the Results section as requested. The Tables are ordered for the outcome variables. Figure 2 provides a visual summary of the associations.

**Reviewer #3**: Finally, why using step-wise regression models, since there are not many independent variables? Why not put all IV in a model and check their significance?

**Author response:** Step-wise regression is preferential to factorial ANOVA as step-wise regression automatically permits forward and backward fitting of variables.

In response to the reviewer’s comment we also add include factorial ANOVA models with all independent variables in a model (Supplemental Table 4). The findings of this were similar to those of step-wise regression.

We copy the additional supplemental data below for ease of reference and comment on these in the revised Results section.

**S4 Table. Results from factorial ANOVA.**

|  | **Number of publications** | **Number of citations** | **Number of citations per publication** | **Average impact factor** |
| --- | --- | --- | --- | --- |
| Student research training degree | 0.8 (P=0.583) | 0.8 (P=0.517) | 1.2 (P=0.293) | 1.2 (P=0.294) |
| Student undergraduate rank | 0.8 (P=0.481) | 0.1 (P=0.938) | 0.5 (P=0.687) | 1.9 (P=0.131) |
| Student academic merit | 2.4 (P=0.068) | 0.7 (P=0.532) | 0.5 (P=0.698) | 2.0 (P=0.111) |
| Student had prior publication | 0.2 (P=0.628) | 0.2 (P=0.698) | 0.0 (P=0.918) | 0.2 (P=0.641) |
| Alignment of research achieved maximum score | 0.0 (P=0.962) | 0.7 (P=0.495) | 1.5 (P=0.230) | 0.4 (P=0.669) |
| Scholarship awarded | **13.4 (P<0.001)** | **6.7 (P=0.010)** | 3.4 (P=0.067) | **10.6 (P=0.001)** |
| Supervisory team achieved maximum score | **4.0 (P=0.047)** | **4.5 (P=0.035)** | 3.5 (P=0.064) | 3.3 (P=0.070) |
| Supervisor in institute or research centre | **4.1 (P=0.045)** | 1.7 (P=0.189) | 2.2 (P=0.142) | 3.2 (P=0.075) |
| Supervisor academic level at application | 0.7 (P=0.533) | 0.4 (P=0.779) | 0.7 (P=0.579) | 0.1 (P=0.972) |

Data are F-value (corresponding P-value). ANOVA fits explanatory variables sequentially to the dependent variables. Explanatory variables were fitted to the dependent variables in the order above (i.e. top variable at left fitted first, followed by the second to top variable).This therefore accounted for potential association of student related factors first to PhD outcomes, with then having a scholarship and then supervisor related factors considered. Despite accounting for student related variables first, having a scholarship and supervisor quality were most consistently associated with outcomes from a student’s PhD.
