# Supplementary material for: Do successful PhD outcomes reflect the research environment rather than academic ability?
Source: PLoS One. 2020 Aug 5;15(8):e0236327. doi: 10.1371/journal.pone.0236327 (PMC7406039; doi:10.1371/journal.pone.0236327)
Supplement: Peer review history

# Peer review: Decision Letter 0

**DOI:** 10.1371/journal.pone.0236327.r001

**Authors:** Cesario Bianchi

31 Oct 2019

PONE-D-19-27758

Are successful PhD outcomes dependent on the research environment or academic ability?

PLOS ONE

Dear Dr. Belavý,

Thank you for submitting your manuscript to PLOS ONE. After careful consideration, we have decided that your manuscript does not meet our criteria for publication and must therefore be rejected.

Specifically:

Unfortunately the manuscript has an heterogeneous student population, lacks the correct statistical analysis and a the small sample size may preclude meaningful conclusions.

I am sorry that we cannot be more positive on this occasion, but hope that you appreciate the reasons for this decision.

Yours sincerely,

Cesario Bianchi

Academic Editor

PLOS ONE

Additional Editor Comments (if provided):

Dear Dr Belavý,

Thank you for submitting your manuscript about an very interesting and important topic. Based on comments of the reviewers and myself I have to reject your present manuscript .

Please, take reviewers 1 comments as a positive criticism to improve your manuscript.

[Note: HTML markup is below. Please do not edit.]

Reviewers' comments:

Reviewer's Responses to Questions

**Comments to the Author**

1. Is the manuscript technically sound, and do the data support the conclusions?

The manuscript must describe a technically sound piece of scientific research with data that supports the conclusions. Experiments must have been conducted rigorously, with appropriate controls, replication, and sample sizes. The conclusions must be drawn appropriately based on the data presented. 

Reviewer #1: No

Reviewer #2: Yes

**********

2. Has the statistical analysis been performed appropriately and rigorously? 

Reviewer #1: No

Reviewer #2: Yes

**********

3. Have the authors made all data underlying the findings in their manuscript fully available?

The PLOS Data policy requires authors to make all data underlying the findings described in their manuscript fully available without restriction, with rare exception (please refer to the Data Availability Statement in the manuscript PDF file). The data should be provided as part of the manuscript or its supporting information, or deposited to a public repository. For example, in addition to summary statistics, the data points behind means, medians and variance measures should be available. If there are restrictions on publicly sharing data—e.g. participant privacy or use of data from a third party—those must be specified.

Reviewer #1: No

Reviewer #2: Yes

**********

4. Is the manuscript presented in an intelligible fashion and written in standard English?

PLOS ONE does not copyedit accepted manuscripts, so the language in submitted articles must be clear, correct, and unambiguous. Any typographical or grammatical errors should be corrected at revision, so please note any specific errors here.

Reviewer #1: Yes

Reviewer #2: Yes

**********

5. Review Comments to the Author

Please use the space provided to explain your answers to the questions above. You may also include additional comments for the author, including concerns about dual publication, research ethics, or publication ethics. (Please upload your review as an attachment if it exceeds 20,000 characters)

Reviewer #1: The present paper covers an important topic, i.e. research policy and selection of PhD students. Very little research has been done on the association between student characteristics, the scientific environment, research output and benefits for society. In this context, the paper covers an important topic and the researchers may have had access to some interesting data.

The study is based on data from one Australian university. Of the 324 applications submitted to the university, 198 students were enrolled in the PhD program, 120 completed the program, 31 are still enrolled, 37 withdraw after starting, and 11 withdrew before starting.

Unfortunately, very few conclusions can be drawn from this small study due to several issues with the statistical analysis and study design.

First of all, the study is a cross-sectional study, and not all students have the same follow-up time. For example, 31 students are still enrolled in the program at the time of the data analysis. Since the inclusion period is from 2010-2013, some students may have been followed for 8 years, others for 4-5 years (please see Rothman KJ. Epidemiology. An Introduction. P. 97. Oxford University Press, 2nd ed., 2012). Moreover, the reader cannot really evaluate the underlying distribution of the data and their validity.

To mention some examples: The mean number of publications was 2.8 with a standard deviation of 4.4; the average impact factor was 1.9 with a standard deviation of 2.36; the mean number of citations per publication was 3.5 with a standard deviation of 7.4; and the total citation was 19.6 with a standard deviation of 49.8.

95% of a normal distribution is to be calculated as mean 1.96 ± standard deviation. This means that if the data were to be correctly described large proportion of students would have negative number of publications, negative impact factor, negative citations, and negative total citations. This does not make sense.

Moreover, it is not clear if this is an etiologic or prediction study (please see Clayton D, Hills M. Statistical Models in Epidemiology, P. 271, Chapter 27, Choice and Interpretation of Models. Oxford University Press, 1993). If it is an etiologic study, a step-wise logistic regression model does not make much sense (please see Rothman KJ. Epidemiology. An Introduction. P. 194. Oxford University Press, 2nd ed., 2012).

This relatively small study has limited statistical precision of the estimates as evident from Table 2. Only significant results have been marked with bold, and many strong associations are ignored simply because they are not statistically significant (please see Amrhein V et al. Scientists rise up against statistical significance. Nature. 2019;567:305-307).

Moreover, the table also shows that the data cannot be described with standard deviation. In addition to lack of statistical power, this type of non-randomized observational study should not focus on statistical significance, but on estimation of the effect (please see Rothman KJ. Six persistent research misconceptions. J Gen Intern Med. 2014;29:1060-4).

Another problem with logistic regression is the rare outcome assumption, which seem to be the case in the present paper (please see Hosmer DW, Lemeshow S, Sturdivant RX. Applied logistic regression, 3rd ed. P. 51. Hoboken: Wiley, 2013).

The study is conducted at a university in Australia as mentioned by the authors, but the external validity should be discussed in more detail.

Overall, the paper covers an important topic, but the study design and the statistical analysis as well as the small sample size make it impossible to draw any valid conclusions.

Reviewer #2: The manuscript that aims to analyzes the PhD students performance based on their previous academic achievement, research environment or supervisor importance is a great piece of work. Basically, the article puts numbers in parameters that the whole scientific community already has an idea, even qualitatively.

Although this is a regionalized study, it can easily extrapolate its findings to other areas of the globe, as the data discussed are important in worldwide PhD programs.

The only suggestion, which may or may not be accepted, would be the inclusion of some graphs, at least for the most significant findings, since understanding the tables is hampered by the number of presented data.

**********

6. PLOS authors have the option to publish the peer review history of their article (what does this mean?). If published, this will include your full peer review and any attached files.

If you choose “no”, your identity will remain anonymous but your review may still be made public.

**Do you want your identity to be public for this peer review?** For information about this choice, including consent withdrawal, please see our Privacy Policy.

Reviewer #1: No

Reviewer #2: No

[NOTE: If reviewer comments were submitted as an attachment file, they will be attached to this email and accessible via the submission site. Please log into your account, locate the manuscript record, and check for the action link "View Attachments". If this link does not appear, there are no attachment files to be viewed.]

- - - - -

For journal use only: PONEDEC3

# Author comment: Author response to Decision Letter 0

**DOI:** 10.1371/journal.pone.0236327.r002

5 Dec 2019

1. Is the manuscript technically sound, and do the data support the conclusions?

Reviewer #1: No

Reviewer #2: Yes

Author response: Reviewers differ in their opinions.

We thank Reviewer #2 for remarking this was ‘a great piece of work’. We also thank both Reviewer #1 and #2 for provided useful ideas. We have responded to the comments of both reviewers and updated the manuscript according. We believe the manuscript to be technically sound and that the data support the conclusions. Notably, as often happens with statistics, especially in larger sample sizes such as here, the different analysis approach did not change the main findings. We believe this contributes to the robustness of the manuscript.

2. Has the statistical analysis been performed appropriately and rigorously?

Reviewer #1: No

Reviewer #2: Yes

Author response: Reviewers differ in their opinions.

We consulted with a professorial biostatistician for the statistical approach during the analysis of the data whilst drafting the original manuscript. 

Reviewer #1 may have missed that we included non-parametric statistics in our analyses. We agree there are concerns using only parametric stats (e.g. assumptions of normality). Therefore, on statistical advice, we included non-parametric statistics. The results of these analyses were essentially the same as those from the parametric stats. This often is the case with larger sample sizes (see Central Limit Theorem). 

Reviewer #2 considered the statistics appropriate in revising the manuscript. We further consulted with the same professorial biostatistician and are confident the statistics are appropriate 

We respond to more specific comments on this point below and have revised the manuscript, accordingly, adding further data Tables and Supplemental Data.

3. Have the authors made all data underlying the findings in their manuscript fully available?

Reviewer #1: No

Reviewer #2: Yes

Author response: Reviewers differ in their opinions.

Our reasons for being unable to provide raw data on the internet were disclosed with the submission. Some of the data are internal confidential university data which could potentially re-identify individual students included in the analyses. The data can be accessed by others with an ethically conform request. 

4. Is the manuscript presented in an intelligible fashion and written in standard English?

Reviewer #1: Yes

Reviewer #2: Yes

Author response: No response required

Reviewer #1

Reviewer #1: The present paper covers an important topic, i.e. research policy and selection of PhD students. Very little research has been done on the association between student characteristics, the scientific environment, research output and benefits for society. In this context, the paper covers an important topic and the researchers may have had access to some interesting data.

Author response: Thank-you for pointing out the novelty and importance of our work.

Reviewer #1: The study is based on data from one Australian university. Of the 324 applications submitted to the university, 198 students were enrolled in the PhD program, 120 completed the program, 31 are still enrolled, 37 withdraw after starting, and 11 withdrew before starting.

Author response: The reviewer reiterates the data sample. No response required. Responses to the reviewer comments based on this summary are given below.

Reviewer #1: Unfortunately, very few conclusions can be drawn from this small study due to several issues with the statistical analysis and study design.

Author response: The data are unique and hard to get at wide scale: (a) key aspects of these data are derived from internal university (i.e. confidential) scholarship ranking panels, (b) universities are typically reluctant to release such information and are highly unlikely to share such data with external investigators due to strict privacy laws, (c) collection of the information is inconsistent between institutions, (d) even within our own university, other Faculties do not collect similar information. Thus, the data are unique.

The ~200 students in the sample are, in light of the granularity and depth of the data, a large sample size. Data involving 1000s of students is difficult to obtain and impossible to obtain at the level of granularity that we have analysed. 

We believe our work is the first on this important topic and the difficulty in attaining the data highlights the uniqueness of our manuscript.

Reviewer #1: First of all, the study is a cross-sectional study, and not all students have the same follow-up time. For example, 31 students are still enrolled in the program at the time of the data analysis. Since the inclusion period is from 2010-2013, some students may have been followed for 8 years, others for 4-5 years (please see Rothman KJ. Epidemiology. An Introduction. P. 97. Oxford University Press, 2nd ed., 2012). Moreover, the reader cannot really evaluate the underlying distribution of the data and their validity.

Author response: We disagree with the Reviewer’s assertion that this is a cross-sectional study. A cross-sectional study is a comparison at a single time-point. In the current study, we retrospectively analysed an entire cohort over 4+ years of follow-up. A cross-sectional study is where all participants are examined at the same time with no follow-up data. 

We painstakingly and carefully combined different data sets, which included checking other internal university databases for whether students changed their name during candidature (as they might publish manuscripts under their new surname, such as after getting married), to ensure we did not miss any one in this cohort.

RE: follow-up period. we thank the reviewer for this comment and agree that some students will have had a longer time to amass publications and citations. We conducted further analyses and student quality and other examined factors did not vary from one year to the next. We have presented this in Supplementary Table 3. 

The parameters that were related to outcomes from PhD were stable over the years considered. Only student undergraduate rank varies over the years depending on year of application. Yet this parameter was unrelated to PhD outcomes. Thus, the longer follow-up period for applicants from 2011 versus those in later years will not impact the findings.

Reviewer #1: To mention some examples: The mean number of publications was 2.8 with a standard deviation of 4.4; the average impact factor was 1.9 with a standard deviation of 2.36; the mean number of citations per publication was 3.5 with a standard deviation of 7.4; and the total citation was 19.6 with a standard deviation of 49.8. 95% of a normal distribution is to be calculated as mean 1.96 ± standard deviation. This means that if the data were to be correctly described large proportion of students would have negative number of publications, negative impact factor, negative citations, and negative total citations. This does not make sense. 

Author response: We thank the reviewer for this comment. We identified concerns with using only parametric stats (e.g. assumptions of normality). Therefore, after consulting with a professorial biostatistician prior to the original submission, we included non-parametric statistics. The results of these analyses were essentially the same as those from the parametric stats. 

We have added a separate table for the non-parametric statistics (presenting medians and interquartile ranges; new Table 2) and retain the original table showing means and standard deviations (Table 3). As stated in the original manuscript, the results are largely the same. This is typical of larger datasets where parametric and non-parametric analyses give largely the same main findings (see Central Limit Theorem).

Notably, we consulted with the same professorial biostatistician for the original submission and this revision.

Reviewer #1: Moreover, it is not clear if this is an etiologic or prediction study (please see Clayton D, Hills M. Statistical Models in Epidemiology, P. 271, Chapter 27, Choice and Interpretation of Models. Oxford University Press, 1993). If it is an etiologic study, a step-wise logistic regression model does not make much sense (please see Rothman KJ. Epidemiology. An Introduction. P. 194. Oxford University Press, 2nd ed., 2012). 

Author response: We sought to examine what baseline (at scholarship ranking) factors were associated with more/less publications, more/less citations and higher/lower impact factor. This is important for choosing (potentially: ‘predicting’) which students are more likely to have the best outcome. 

Our work would then fall under the reviewer’s ‘prediction’ category, making the criticism of step-wise logistic regression not applicable. 

We consulted with a professorial biostatistician for the original submission and this revision, hence we are confident that these statistics have been conducted appropriately.

Reviewer #1: This relatively small study has limited statistical precision of the estimates as evident from Table 2. 

Author response: As mentioned prior:

The data are unique and hard to get at wide scale: (a) key aspects of these data are derived from internal university (i.e. confidential) scholarship ranking panels, (b) universities are typically reluctant to release such information and are highly unlikely to share such data with external investigators due to strict privacy laws, (c) collection of the information is inconsistent between institutions, (d) even within our own university, other Faculties do not collect similar information. Thus, the data are unique.

The ~200 students in the sample are, in light of the granularity and depth of the data, a large sample size. Data involving 1000s of students is difficult to obtain and impossible to obtain at the level of granularity that we have analysed. 

We believe our work is the first on this important topic and the difficulty in attaining the data highlights the uniqueness of our manuscript.

Reviewer #1: Only significant results have been marked with bold, and many strong associations are ignored simply because they are not statistically significant (please see Amrhein V et al. Scientists rise up against statistical significance. Nature. 2019;567:305-307). 

Author response: In the revised manuscript, we presented effect sizes (see new Figure 2 and Supplemental Tables 1 and 2). As often happens in statistical analyses, the statistical significance is associated with the largest effect sizes. 

Aside from statistically significant effects, we also included in the Discussion (paragraph 1) consideration of effect sizes.

Notably, we consulted with a professorial biostatistician for the original submission and this revision.

Reviewer #1: Moreover, the table also shows that the data cannot be described with standard deviation. 

Author response: As mentioned prior: 

We have added a separate table for the non-parametric statistics (presenting medians and interquartile ranges; new Table 2) and retain the original table showing means and standard deviations (Table 3). As stated in the original manuscript, the results are largely the same. This is typical of larger datasets where parametric and non-parametric analyses give largely the same main findings (see Central Limit Theorem).

Reviewer #1: Another problem with logistic regression is the rare outcome assumption, which seem to be the case in the present paper (please see Hosmer DW, Lemeshow S, Sturdivant RX. Applied logistic regression, 3rd ed. P. 51. Hoboken: Wiley, 2013).

Author response: This comment refers to the drop-out of students from PhD. 

In our sample 19.7% of students dropped out of PhD at the Government’s census cut-off date. We posit that 20% is a frequent event rather than a ‘rare outcome’. However, this same topic also refers to the sample size and potential for bias in the logit coefficients (King & Zeng "Logistic Regression in Rare Events Data". Political Analysis, 2001. 9: 137-163; https://gking.harvard.edu/files/0s.pdf). We take on this consideration.

In the revised manuscript we implement the Penalized Maximum Likelihood Estimation proposed by Firth ("Bias reduction of maximum likelihood estimates" Biometrika 80:27-38; https://doi.org/10.1093/biomet/80.1.27). This is considered the best approach to implementing logistic regression in sample sizes such as in the current study.

The findings of the logistic regression analyses are similar to those presented in the original manuscript. 

Reviewer #1: In addition to lack of statistical power, this type of non-randomized observational study should not focus on statistical significance, but on estimation of the effect (please see Rothman KJ. Six persistent research misconceptions. J Gen Intern Med. 2014;29:1060-4).

Author response: As mentioned prior:

RE: Sample size:

The data are unique and hard to get at wide scale: (a) key aspects of these data are derived from internal university (i.e. confidential) scholarship ranking panels, (b) universities are typically reluctant to release such information and are highly unlikely to share such data with external investigators due to strict privacy laws, (c) collection of the information is inconsistent between institutions, (d) even within our own university, other Faculties do not collect similar information. Thus, the data are unique.

The ~200 students in the sample are, in light of the granularity and depth of the data, a large sample size. Data involving 1000s of students is difficult to obtain and impossible to obtain at the level of granularity that we have analysed. 

We believe our work is the first on this important topic and the difficulty in attaining the data highlights the uniqueness of our manuscript.

RE: effect sizes:

In the revised manuscript, we presented effect sizes (see new Figure 2 and Supplemental Tables 1 and 2). As often happens in statistical analyses, the statistical significance is associated with the largest effect sizes. 

Aside from statistically significant effects, we also included in the Discussion (paragraph 1) consideration of effect sizes.

Notably, we consulted with a professorial biostatistician for the original submission and this revision.

Reviewer #1: The study is conducted at a university in Australia as mentioned by the authors, but the external validity should be discussed in more detail.

Author response: As mentioned prior:

RE: Sample size:

The data are unique and hard to get at wide scale: (a) key aspects of these data are derived from internal university (i.e. confidential) scholarship ranking panels, (b) universities are typically reluctant to release such information and are highly unlikely to share such data with external investigators due to strict privacy laws, (c) collection of the information is inconsistent between institutions, (d) even within our own university, other Faculties do not collect similar information. Thus, the data are unique.

The ~200 students in the sample are, in light of the granularity and depth of the data, a large sample size. Data involving 1000s of students is difficult to obtain and impossible to obtain at the level of granularity that we have analysed. 

We believe our work is the first on this important topic and the difficulty in attaining the data highlights the uniqueness of our manuscript.

Furthermore, we discuss this further in the Discussion (2nd last paragraph). 

Notably, Reviewer #2 stated ‘Although this is a regionalized study, it can easily extrapolate its findings to other areas of the globe’.

Reviewer #1: Overall, the paper covers an important topic, but the study design and the statistical analysis as well as the small sample size make it impossible to draw any valid conclusions.

Author response: We agree this is an important topic. We think the reviewer had some very good points. In light of the Reviewer’s comments, we have revised the manuscript and believe that it is much stronger as a consequence. 

Reviewer #2

Reviewer #2: The manuscript that aims to analyzes the PhD students performance based on their previous academic achievement, research environment or supervisor importance is a great piece of work. Basically, the article puts numbers in parameters that the whole scientific community already has an idea, even qualitatively.

Author response: Thank-you for your positive comments on our manuscript. 

Reviewer #2: Although this is a regionalized study, it can easily extrapolate its findings to other areas of the globe, as the data discussed are important in worldwide PhD programs.

Author response: We agree with the comment. We discuss this further in the Discussion (2nd last paragraph).

Reviewer #2: The only suggestion, which may or may not be accepted, would be the inclusion of some graphs, at least for the most significant findings, since understanding the tables is hampered by the number of presented data.

Author response: Thank-you for this suggestion. In the revised manuscript we include a new Figure 2 which displays the effect sizes of the main findings.

Attachment Submitted filename: 2019_12_05_PONE-D-19-27758_Response.docx Click here for additional data file.

# Peer review: Decision Letter 1

**DOI:** 10.1371/journal.pone.0236327.r003

**Authors:** Sergi Lozano

21 Apr 2020

PONE-D-19-27758R1

Successful PhD outcomes are dependent on the research environment and not academic ability

PLOS ONE

Dear Dr. Belavý,

Thank you for submitting your manuscript to PLOS ONE. After careful consideration, we feel that it has merit but does not fully meet PLOS ONE’s publication criteria as it currently stands. Therefore, we invite you to submit a revised version of the manuscript that addresses the points raised during the review process.

Notice that, in this round, a third reviewer has been invited to have a more complete assessment of your work.

When revising the article, please pay special attention to methodological concerns by both Reviewers #1 and #3. The comment made by Reviewer 2 regarding the title should also be considered.

We would appreciate receiving your revised manuscript by Jun 05 2020 11:59PM. When you are ready to submit your revision, log on to https://www.editorialmanager.com/pone/ and select the 'Submissions Needing Revision' folder to locate your manuscript file.

If you would like to make changes to your financial disclosure, please include your updated statement in your cover letter.

To enhance the reproducibility of your results, we recommend that if applicable you deposit your laboratory protocols in protocols.io, where a protocol can be assigned its own identifier (DOI) such that it can be cited independently in the future. For instructions see: http://journals.plos.org/plosone/s/submission-guidelines#loc-laboratory-protocols

Please include the following items when submitting your revised manuscript:

- A rebuttal letter that responds to each point raised by the academic editor and reviewer(s). This letter should be uploaded as separate file and labeled 'Response to Reviewers'.
- A marked-up copy of your manuscript that highlights changes made to the original version. This file should be uploaded as separate file and labeled 'Revised Manuscript with Track Changes'.
- An unmarked version of your revised paper without tracked changes. This file should be uploaded as separate file and labeled 'Manuscript'.

Please note while forming your response, if your article is accepted, you may have the opportunity to make the peer review history publicly available. The record will include editor decision letters (with reviews) and your responses to reviewer comments. If eligible, we will contact you to opt in or out.

We look forward to receiving your revised manuscript.

Kind regards,

Sergi Lozano

Filippo Radicchi, Ph.D.

Academic Editors

PLOS ONE

Journal Requirements:

1. Please ensure that your manuscript meets PLOS ONE's style requirements, including those for file naming. The PLOS ONE style templates can be found at

http://www.journals.plos.org/plosone/s/file?id=wjVg/PLOSOne_formatting_sample_main_body.pdf and http://www.journals.plos.org/plosone/s/file?id=ba62/PLOSOne_formatting_sample_title_authors_affiliations.pdf

2. We note that you have indicated that data from this study are available upon request. PLOS only allows data to be available upon request if there are legal or ethical restrictions on sharing data publicly. For more information on unacceptable data access restrictions, please see http://journals.plos.org/plosone/s/data-availability#loc-unacceptable-data-access-restrictions.

In your revised cover letter, please address the following prompts:

a) If there are ethical or legal restrictions on sharing a de-identified data set, please explain them in detail (e.g., data contain potentially sensitive information, data are owned by a third-party organization, etc.) and who has imposed them (e.g., an ethics committee). Please also provide contact information for a data access committee, ethics committee, or other institutional body to which data requests may be sent.

b) If there are no restrictions, please upload the minimal anonymized data set necessary to replicate your study findings as either Supporting Information files or to a stable, public repository and provide us with the relevant URLs, DOIs, or accession numbers. For a list of acceptable repositories, please see http://journals.plos.org/plosone/s/data-availability#loc-recommended-repositories.

We will update your Data Availability statement on your behalf to reflect the information you provide.

3. Please remove your figures from within your manuscript file, leaving only the individual TIFF/EPS image files, uploaded separately. These will be automatically included in the reviewers’ PDF.

4. We note you have included a table to which you do not refer in the text of your manuscript. Please ensure that you refer to Table 5 in your text; if accepted, production will need this reference to link the reader to the Table.

5. Please include captions for your Supporting Information files at the end of your manuscript, and update any in-text citations to match accordingly. Please see our Supporting Information guidelines for more information: http://journals.plos.org/plosone/s/supporting-information.

6. Thank you for including your ethics statement on the online submission form: "Deakin University Human Research Ethics Committee reviewed this project (2019-191) and found it to be compliant with the Ethical Considerations in Quality Assurance and Evaluation Activities guidelines of the National Health and Medical Research Council of Australia and determined that no further ethics review was required. Consent was not obtained (the data were analyzed anonymously). ".

To help ensure that the wording of your manuscript is suitable for publication, would you please also add this statement at the beginning of the Methods section of your manuscript file

[Note: HTML markup is below. Please do not edit.]

Reviewers' comments:

Reviewer's Responses to Questions

**Comments to the Author**

1. If the authors have adequately addressed your comments raised in a previous round of review and you feel that this manuscript is now acceptable for publication, you may indicate that here to bypass the “Comments to the Author” section, enter your conflict of interest statement in the “Confidential to Editor” section, and submit your "Accept" recommendation.

Reviewer #1: (No Response)

Reviewer #2: All comments have been addressed

Reviewer #3: (No Response)

**********

2. Is the manuscript technically sound, and do the data support the conclusions?

The manuscript must describe a technically sound piece of scientific research with data that supports the conclusions. Experiments must have been conducted rigorously, with appropriate controls, replication, and sample sizes. The conclusions must be drawn appropriately based on the data presented. 

Reviewer #1: No

Reviewer #2: Yes

Reviewer #3: No

**********

3. Has the statistical analysis been performed appropriately and rigorously? 

Reviewer #1: No

Reviewer #2: Yes

Reviewer #3: No

**********

4. Have the authors made all data underlying the findings in their manuscript fully available?

The PLOS Data policy requires authors to make all data underlying the findings described in their manuscript fully available without restriction, with rare exception (please refer to the Data Availability Statement in the manuscript PDF file). The data should be provided as part of the manuscript or its supporting information, or deposited to a public repository. For example, in addition to summary statistics, the data points behind means, medians and variance measures should be available. If there are restrictions on publicly sharing data—e.g. participant privacy or use of data from a third party—those must be specified.

Reviewer #1: No

Reviewer #2: Yes

Reviewer #3: No

**********

5. Is the manuscript presented in an intelligible fashion and written in standard English?

PLOS ONE does not copyedit accepted manuscripts, so the language in submitted articles must be clear, correct, and unambiguous. Any typographical or grammatical errors should be corrected at revision, so please note any specific errors here.

Reviewer #1: No

Reviewer #2: Yes

Reviewer #3: Yes

**********

6. Review Comments to the Author

Please use the space provided to explain your answers to the questions above. You may also include additional comments for the author, including concerns about dual publication, research ethics, or publication ethics. (Please upload your review as an attachment if it exceeds 20,000 characters)

Reviewer #1: Thank you very much for giving me the opportunity to review the revised paper and the authors’ response.

The study focuses on an important topic, but the authors have only responded to the reviewer comments to a limited extent. Their main reply is that their data are unique and that they have

consulted a statistician. However, the manuscript still has several issues.

The material consists of fewer than 200 persons and the authors have conducted several cross-sectional analyses that the authors did not critically consider.

There is a large number of p-values in the manuscript yet the authors did not give any thought to statistical power in this small study population.

Moreover, the world’s leading and prominent scientists warned against this type of analysis, that is, statistical significance testing, in which only significant results are highligted. Please see Nature: https://www.nature.com/articles/d41586-019-00857-9

For this study to be a cohort analysis, the persons should either have had the same follow-up time, or the authors should have taken the various follow-up periods into consideration in the statistical analysis. This is not the case. The present analysis is solely cross-sectional.

It seems unclear to the authors if they have conducted a prediction study, or a causal study. It seems as if they tend to believe that their study is more of a causal study than a prediction study.

It is furthermore not clear what the authors want to estimate by use of odds ratios – is it prevalence rate ratio, incidence rate ratio, or relative risks? The study design does not meet the criteria for the latter two.

It still does not make any sense to report variables by mean and standard deviation.

Reviewer #2: Since the first submission, I found the manuscript interesting. Few studies, even if regionalized like this, show so clearly the importance of the group in which the student is inserted.

However, the title may sound aggressive in the way it is written. My opinion is that the words could be less impactful in the title...even if the results clearly show the importance of the group in the researcher formation

Reviewer #3: The manuscript studies factors related to PhD outcome. This is an important topic. However, my primary concern is that the manuscript has made strong claims that were not supported by the analysis.

For starters, in the economics of education literature, there are essentially two schools of thought: (1) education signals how good a student is, irrespective of the quality of the training; and (2) education provides training. It is important to disentangle the two effects, and there have been a lot of studies trying to do this. Therefore, I am not convinced that “To the best of our knowledge, this is the first analysis of PhD student outcomes in relation to their research environment, their academic abilities and prior research training.” The authors may want to do more literature search on this topic.

A related comment is that, in the paper’s context, the fact that some students are able to do research in a strategic research center and their supervisory teams who got maximum scores may already signal that they may have better academic abilities, thus have better outcomes, as observed in the paper. In other words, it is academic abilities that affect PhD outcome.

It remains unclear to me what the operational definitions of “research environment” and “academic ability” are. Which variables fall into which category? Does scholarship reflect academic ability? If so, the observation that students who are awarded scholarships have more papers/citations and are less likely to withdraw from PhD directly refuted the major claim of the paper.

There are two variables that indicate whether strategic alignment score and supervisor team score achieve maximum. Why focusing on maximum? Wouldn’t a maximum score emphasize the very best?

The current of flow in the results section is quite confusing, alternating between different variables and outcomes. I’d suggest focusing on one outcome at a time and for each outcome describing univariate analysis and regression analysis. A summary table that indicates the associations of each independent variable and each outcome is also helpful.

Finally, why using step-wise regression models, since there are not many independent variables? Why not put all IV in a model and check their significance?

**********

7. PLOS authors have the option to publish the peer review history of their article (what does this mean?). If published, this will include your full peer review and any attached files.

If you choose “no”, your identity will remain anonymous but your review may still be made public.

**Do you want your identity to be public for this peer review?** For information about this choice, including consent withdrawal, please see our Privacy Policy.

Reviewer #1: No

Reviewer #2: No

Reviewer #3: No

[NOTE: If reviewer comments were submitted as an attachment file, they will be attached to this email and accessible via the submission site. Please log into your account, locate the manuscript record, and check for the action link "View Attachments". If this link does not appear, there are no attachment files to be viewed.]

While revising your submission, please upload your figure files to the Preflight Analysis and Conversion Engine (PACE) digital diagnostic tool, https://pacev2.apexcovantage.com/. PACE helps ensure that figures meet PLOS requirements. To use PACE, you must first register as a user. Registration is free. Then, login and navigate to the UPLOAD tab, where you will find detailed instructions on how to use the tool. If you encounter any issues or have any questions when using PACE, please email us at figures@plos.org. Please note that Supporting Information files do not need this step.

# Author comment: Author response to Decision Letter 1

**DOI:** 10.1371/journal.pone.0236327.r004

19 May 2020

Please see Response to Reviewers file submitted with manuscript

Attachment Submitted filename: 2020_05_19_PONE-D-19-27758R2_Response.docx Click here for additional data file.

# Peer review: Decision Letter 2

**DOI:** 10.1371/journal.pone.0236327.r005

**Authors:** Sergi Lozano

7 Jul 2020

Do successful PhD outcomes reflect the research environment rather than academic ability?

PONE-D-19-27758R2

Dear Dr. Belavý,

We’re pleased to inform you that your manuscript has been judged scientifically suitable for publication and will be formally accepted for publication once it meets all outstanding technical requirements.

Within one week, you’ll receive an e-mail detailing the required amendments. When these have been addressed, you’ll receive a formal acceptance letter and your manuscript will be scheduled for publication.

An invoice for payment will follow shortly after the formal acceptance. To ensure an efficient process, please log into Editorial Manager at http://www.editorialmanager.com/pone/, click the 'Update My Information' link at the top of the page, and double check that your user information is up-to-date. If you have any billing related questions, please contact our Author Billing department directly at authorbilling@plos.org.

If your institution or institutions have a press office, please notify them about your upcoming paper to help maximize its impact. If they’ll be preparing press materials, please inform our press team as soon as possible -- no later than 48 hours after receiving the formal acceptance. Your manuscript will remain under strict press embargo until 2 pm Eastern Time on the date of publication. For more information, please contact onepress@plos.org.

Kind regards,

Sergi Lozano

Academic Editor

PLOS ONE

Additional Editor Comments (optional):

Reviewers' comments:

Reviewer's Responses to Questions

**Comments to the Author**

1. If the authors have adequately addressed your comments raised in a previous round of review and you feel that this manuscript is now acceptable for publication, you may indicate that here to bypass the “Comments to the Author” section, enter your conflict of interest statement in the “Confidential to Editor” section, and submit your "Accept" recommendation.

Reviewer #3: All comments have been addressed

**********

2. Is the manuscript technically sound, and do the data support the conclusions?

The manuscript must describe a technically sound piece of scientific research with data that supports the conclusions. Experiments must have been conducted rigorously, with appropriate controls, replication, and sample sizes. The conclusions must be drawn appropriately based on the data presented. 

Reviewer #3: (No Response)

**********

3. Has the statistical analysis been performed appropriately and rigorously? 

Reviewer #3: (No Response)

**********

4. Have the authors made all data underlying the findings in their manuscript fully available?

The PLOS Data policy requires authors to make all data underlying the findings described in their manuscript fully available without restriction, with rare exception (please refer to the Data Availability Statement in the manuscript PDF file). The data should be provided as part of the manuscript or its supporting information, or deposited to a public repository. For example, in addition to summary statistics, the data points behind means, medians and variance measures should be available. If there are restrictions on publicly sharing data—e.g. participant privacy or use of data from a third party—those must be specified.

Reviewer #3: (No Response)

**********

5. Is the manuscript presented in an intelligible fashion and written in standard English?

PLOS ONE does not copyedit accepted manuscripts, so the language in submitted articles must be clear, correct, and unambiguous. Any typographical or grammatical errors should be corrected at revision, so please note any specific errors here.

Reviewer #3: (No Response)

**********

6. Review Comments to the Author

Please use the space provided to explain your answers to the questions above. You may also include additional comments for the author, including concerns about dual publication, research ethics, or publication ethics. (Please upload your review as an attachment if it exceeds 20,000 characters)

Reviewer #3: (No Response)

**********

7. PLOS authors have the option to publish the peer review history of their article (what does this mean?). If published, this will include your full peer review and any attached files.

If you choose “no”, your identity will remain anonymous but your review may still be made public.

**Do you want your identity to be public for this peer review?** For information about this choice, including consent withdrawal, please see our Privacy Policy.

Reviewer #3: No

# Editor report: Acceptance letter

**DOI:** 10.1371/journal.pone.0236327.r006

**Authors:** Sergi Lozano

21 Jul 2020

PONE-D-19-27758R2 

Do successful PhD outcomes reflect the research environment rather than academic ability? 

Dear Dr. Belavy:

I'm pleased to inform you that your manuscript has been deemed suitable for publication in PLOS ONE. Congratulations! Your manuscript is now with our production department. 

If your institution or institutions have a press office, please let them know about your upcoming paper now to help maximize its impact. If they'll be preparing press materials, please inform our press team within the next 48 hours. Your manuscript will remain under strict press embargo until 2 pm Eastern Time on the date of publication. For more information please contact onepress@plos.org.

If we can help with anything else, please email us at plosone@plos.org. 

Thank you for submitting your work to PLOS ONE and supporting open access. 

Kind regards, 

PLOS ONE Editorial Office Staff

on behalf of

Dr. Sergi Lozano 

Academic Editor

PLOS ONE
